# Progressive transformation of the HIV-1 reservoir cell profile over two decades of antiviral therapy

**DOI:** 10.1016/j.chom.2022.12.002

**Published:** 2023-01-11

**Authors:** Xiaodong Lian, Kyra W. Seiger, Elizabeth M. Parsons, Ce Gao, Weiwei Sun, Gregory T. Gladkov, Isabelle C. Roseto, Kevin B. Einkauf, Matthew R. Osborn, Joshua M. Chevalier, Chenyang Jiang, Jane Blackmer, Mary Carrington, Eric S. Rosenberg, Michael M. Lederman, Deborah K. McMahon, Ronald J. Bosch, Jeffrey M. Jacobson, Rajesh T. Gandhi, Michael J. Peluso, Tae-Wook Chun, Steven G. Deeks, Xu G. Yu, Mathias Lichterfeld

**Affiliations:** 1 Infectious Disease Division, Brigham and Women’s Hospital, Boston, MA 02115, USA; 2 Ragon Institute of MGH, MIT and Harvard, Cambridge, MA 02139, USA; 3 Basic Science Program, Frederick National Laboratory for Cancer Research, Frederick, MD 21701, USA; 4 Infectious Disease Division, Massachusetts General Hospital, Boston, MA 02114, USA; 5 Case Western Reserve University, Cleveland, OH 44106, USA; 6 University of Pittsburgh, Pittsburgh, PA 15260, USA; 7 Center for Biostatistics in AIDS Research, Harvard T.H. Chan School of Public Health, Boston, MA 02115, USA; 8 Division of HIV, Infectious Diseases and Global Medicine, University of California San Francisco, San Francisco, CA 94143, USA; 9 Laboratory of Immunoregulation, National Institute of Allergy and Infectious Diseases (NIAID), National Institutes of Health (NIH), Bethesda, MD 20892, USA; 10 Laboratory of Integrative Cancer Immunology, Center for Cancer Research, National Cancer Institute, Bethesda, MD 20892, USA

**Keywords:** HIV, latency, integration sites, post-treatment controllers, HIV eradication, FLIP-seq, MIP-seq, HIV cure, block and lock, retroviral pathogenesis

## Abstract

HIV-1 establishes a life-long reservoir of virally infected cells which cannot be eliminated by antiretroviral therapy (ART). Here, we demonstrate a markedly altered viral reservoir profile of long-term ART-treated individuals, characterized by large clones of intact proviruses preferentially integrated in heterochromatin locations, most prominently in centromeric satellite/micro-satellite DNA. Longitudinal evaluations suggested that this specific reservoir configuration results from selection processes that promote the persistence of intact proviruses in repressive chromatin positions, while proviruses in permissive chromosomal locations are more likely to be eliminated. A bias toward chromosomal integration sites in heterochromatin locations was also observed for intact proviruses in study participants who maintained viral control after discontinuation of antiretroviral therapy. Together, these results raise the possibility that antiviral selection mechanisms during long-term ART may induce an HIV-1 reservoir structure with features of deep latency and, possibly, more limited abilities to drive rebound viremia upon treatment interruptions.

## Highlights

• After LT-ART, intact HIV proviruses are predominantly integrated in heterochromatin
• These distinct integration sites result from longitudinal selection mechanisms
• No similar selection processes are observed for defective proviruses
• Intact proviruses are mainly integrated in heterochromatin in post-treatment controllers

## Graphical abstract

**Figure:**
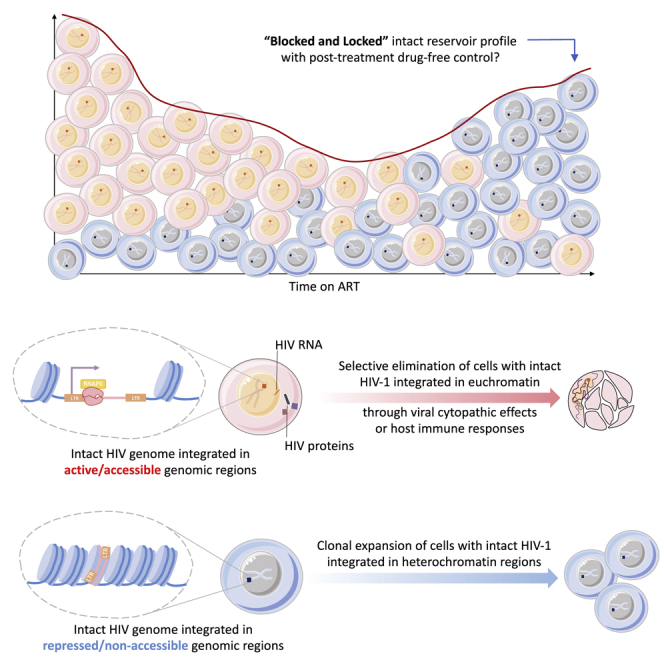

## Teaser

Lian et al. show that following two decades of continuous antiretroviral therapy, the integration site profile of intact HIV-1 proviruses is heavily biased toward heterochromatin locations, likely as a result of immune selection mechanisms; such proviruses are less transcriptionally active and, possibly, less rebound competent.

## Introduction

Antiretroviral combination therapy is highly effective in suppressing HIV-1 replication and is recommended as the standard of care for all people living with HIV (PLHIV). Through the inhibition of HIV-1 reverse transcriptase, integrase, or protease, antiretroviral drugs can effectively prevent infection of new viral target cells; however, they have no direct activity against already infected cells, some of which persist as a long-term viral reservoir and make HIV-1 infection an incurable disease.^1^^,^^2^^,^^3^ These cells have traditionally been considered as “latently infected” lymphocytes that harbor “transcriptionally silent” proviruses,^4^ but recent results have shown that a significant proportion of these cells can remain transcriptionally active,^5^^,^^6^^,^^7^^,^^8^ consistent with the fact that antiretroviral agents do not directly inhibit viral gene transcription from chromosomal DNA. Ongoing viral gene transcription and protein translation may make HIV-1-infected cells visible to immune cells and increase their vulnerability to host-dependent immune activity or viral cytopathic effects; in contrast, true viral latency, defined by extremely limited or no residual viral transcriptional activity, can be viewed as an effective strategy to escape from host immune surveillance. Recent work suggested that in persons with natural control of HIV-1 (termed elite controllers [ECs]) the majority of genome-intact proviral sequences were integrated in heterochromatin locations that do not support effective viral transcription—a state that we and others have termed “deep latency.”^9^^,^^10^^,^^11^ We proposed that such a distinct viral reservoir profile, colloquially referred to as a “block and lock” pattern, is the result of immune-mediated selection mechanisms that preferentially eliminate proviruses located in chromosomal positions more supportive of proviral transcriptional activity, while intact proviruses in repressive chromatin locations persist long term. A proviral reservoir landscape dominated by intact proviruses in heterochromatin positions may only be poorly rebound-competent *in vivo* and is likely to contribute to the remarkable ability of ECs to maintain a durable, drug-free remission of HIV-1 infection. In this work, we hypothesized that selection of viral reservoir cells with features of deep viral latency may also occur in antiretroviral therapy (ART)-treated individuals, although presumably with weaker efficacy than in ECs. Reasoning that extended durations of suppressive ART may allow such selection forces to become more visible, we here conducted a detailed analysis of the HIV-1 reservoir profile for a group of persons who remained on suppressive ART for approximately two decades.

## Results

### Frequencies of intact proviruses in persons undergoing long-term ART

**Figure 1:**
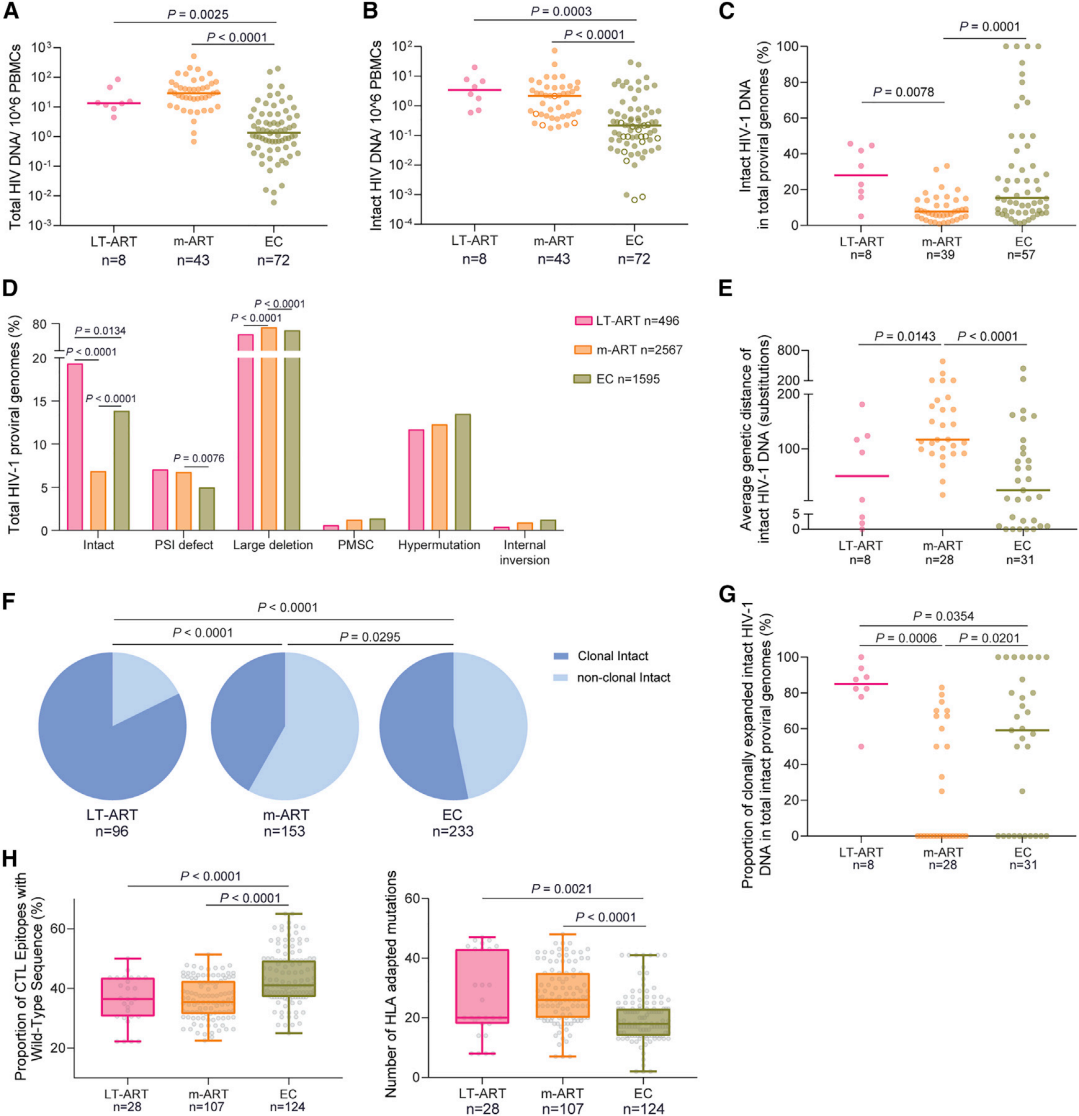
Proviral reservoir profile in long-term ART-treated individuals (A and B) Frequency of total (A) and intact (B) HIV-1 proviruses in long-term ART-treated individuals (LT-ART), in people living with HIV (PLHIV) undergoing moderate durations of treatment (median of 9 years) (m-ART), and in elite controllers (ECs). Open circles indicate data at the limit of detection. (C) Proportion of intact proviruses within total HIV-1 proviruses in indicated study cohorts. (D) Proportion of proviruses with genome-intact or defective sequences in indicated study cohorts. (E) Average genetic distance between intact proviruses from indicated study cohorts, determined by pairwise comparisons between all intact proviruses from a given study participant. (F) Pie charts reflecting proportions of intact proviruses detected once (non-clonal) or multiple times (clonal) in the three indicated study cohorts. (G) Proportions of clonally expanded intact HIV-1 proviruses within the total pool of intact HIV-1-proviruses from each study participant. (H) (Left panel) Proportion of wild-type clade B CTL epitopes restricted by autologous HLA class I alleles within intact proviruses from indicated study cohorts. Each symbol represents one intact proviral sequence; all intact clade B sequences were included. (Right panel) Numbers of base pair variations significantly associated with autologous HLA class I alleles, determined as described by Carlson et al.,^13^ within intact HIV-1 proviruses from indicated study participants. Each symbol reflects one intact provirus. Clonal sequences were counted once. Box and Whisker plots demonstrate median, interquartile ranges, and minimum/maximum. (A–H) FDR-adjusted two-sided Kruskal-Wallis nonparametric test or FDR-adjusted Fisher’s exact test were used, as appropriate. (A–C, E, and G) Horizontal bars indicate the median and n represents the number of study subjects. (D, F, and H) n reflects the number of viral sequences.

We evaluated HIV-1 reservoir cells in study participants (n = 8) who stayed on continuous suppressive ART for a median of 20 (range: 18–23) consecutive years (with no more than 2 recorded plasma viremia blips [<100 copies/mL]) and were among the first generation of PLHIV benefiting from the roll-out of triple-drug antiretroviral combination therapy from 1996 to 2000 (Figure S1). For an initial characterization of the HIV-1 reservoir profile in these long-term ART-treated individuals (LT-ART), we subjected their peripheral blood mononuclear cells (PBMC) to near full-length individual proviral sequencing (FLIP-seq), a protocol that relies on single-genome next-generation sequencing of HIV-1 proviral DNA, followed by biocomputational identification of genome-intact proviruses defined by the absence of lethal sequence variations.^12^ These studies, involving a total of 496 individual proviral genomes from LT-ART individuals (n = 96 intact and n = 400 defective proviruses), demonstrated no specific difference in the frequency of genome-intact proviral sequences in LT-ART individuals relative to persons with moderate durations of suppressive ART exposure (median of 9 years) (m-ART). In comparison with ECs, the frequencies of intact proviruses in LT-ART study participants were significantly higher (Figures 1A and 1B). However, the ratio of genome-intact proviruses to defective proviruses (Figures 1C and 1D), and the average intra-individual genetic distance between intact proviruses (Figure 1E), were more similar between LT-ART individuals and ECs, relative to persons with moderate ART treatment durations. Phylogenetic evaluations indicated that large proportions of intact proviruses from LT-ART individuals were part of sequence-identical clonal clusters that derive from clonal proliferation of virally infected cells; the proportion of intact proviruses that were part of such large clones was highest in LT-ART individuals (Figures 1F and 1G), although such large clones of intact proviruses were also evident in ECs. Notably, clusters of sequence-identical genome-intact or replication-competent HIV-1 proviruses have also been observed in persons with more limited durations of ART; however, the general landscape of intact proviruses in such individuals was more diverse.^14^^,^^15^^,^^16^^,^^17^^,^^18^^,^^19^ The number of HLA-class-I-associated sequence variations in intact proviruses did not differ profoundly between LT-ART individuals and ART-treated PLHIV with moderate treatment durations, and was lowest in ECs (Figures 1H and S2).

### Persistence of intact proviruses integrated in centromeres and in satellite DNA

**Figure 2:**
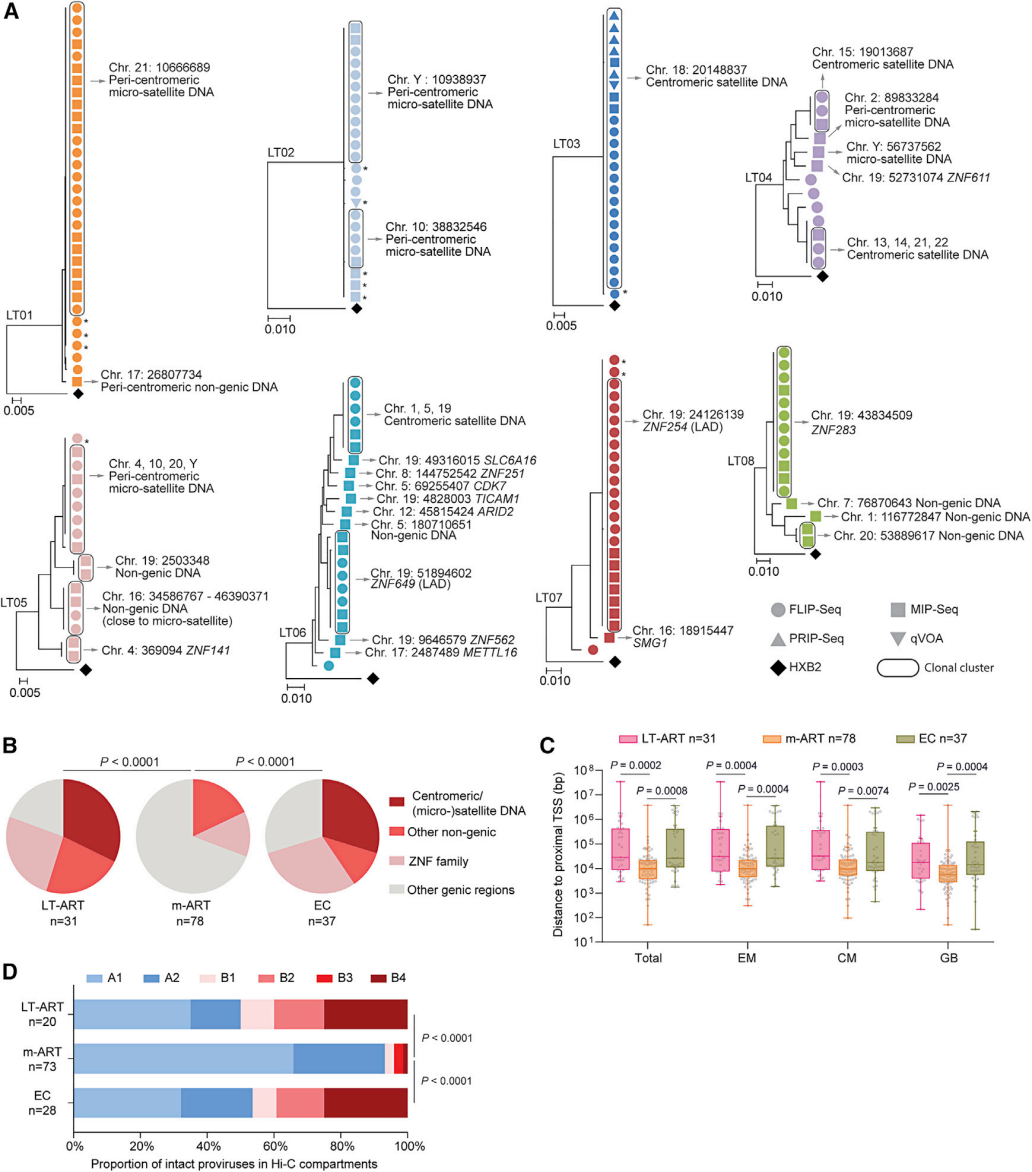
Chromosomal positioning of intact HIV-1 proviruses in long-term ART-treated individuals (A) Maximum-likelihood phylogenetic trees for intact HIV-1 proviruses from long-term ART-treated individuals (LT01–LT08). Coordinates of chromosomal integration sites obtained by integration site loop amplification (ISLA) and corresponding gene name (if applicable) are indicated. Symbols indicate sequences generated by FLIP-seq, MIP-seq, PRIP-seq or from quantitative viral outgrowth assays (qVOAs). Proviruses integrated in highly repetitive satellite DNA could not always be definitively mapped to specific chromosomal locations; a detailed list of integration sites is shown in Table S1. ^∗^Sequences differ by 1 or 2 base pairs from adjacent clonal sequences. LADs, lamina-associated domains. (B) Proportions of intact proviruses with indicated integration site features in LT-ART individuals and comparison cohorts. (C) Chromosomal distance between integration sites of intact proviruses to most proximal host transcriptional start sites (TSSs), as determined by RNA-seq in CD4 T cells from reference datasets in total, effector-memory (EM), or central-memory (CM) primary CD4 T cells or from genome browser (GB).^16^ Box and Whisker plots show median, interquartile ranges and minimum/maximum. (D) Proportions of genome-intact proviral sequences in structural compartments/subcompartments A and B, as determined by Hi-C seq data.^24^ Integration sites not covered in the reference dataset were excluded. (B–D) Data from individuals with moderate ART treatment durations (m-ART) and from EC are shown for comparison. Clonal sequences are counted once. (B–D) p values were calculated by FDR-adjusted two-sided Kruskal-Wallis nonparametric tests or chi-square tests, adjusted for multiple comparison testing where applicable. n reflects the number of integration sites.

To profile the chromosomal locations of intact proviruses, we used “matched integration site and proviral sequencing” (MIP-seq), an approach that focuses on phi29-catalyzed multiple displacement amplification of individual proviruses, followed by paired assessments of proviral sequences and corresponding proviral chromosomal integration sites.^16^ In our cross-sectional analysis, we identified independent integration sites of n = 31 intact proviruses from LT-ART individuals. Most notably, these studies revealed that integration sites of intact proviruses from LT-ART individuals were profoundly biased toward centromeric satellite DNA, peri-centromeric micro-satellite DNA, or non-genic DNA, which generally represent disfavored targets for proviral integration during acute HIV-1 infection,^20^^,^^21^ and can confer a state of deep latency^8^^,^^22^^,^^23^ (Figures 2A and S3; Tables S1 and S2). In study participants LT01–03, the proviral reservoir landscape consisted almost exclusively of large clones of genome-intact proviruses integrated in the centromeric or peri-centromeric satellite/micro-satellite DNA of chromosome 21 (participant LT01), chromosome 10 and chromosome Y (participant LT02), or chromosome 18 (participant LT03). In study participants LT04–06, a more oligoclonal viral reservoir profile was observed; however, large clones of genome-intact proviruses integrated in satellite DNA were equally visible in these participants. Further, some intact proviral genomes in these three participants were integrated in highly repetitive satellite DNA regions that were present on multiple chromosomes and could not be definitively mapped to one specific chromosomal site. Notably, in study participant LT04, the integration of an intact provirus into non-centromeric micro-satellite DNA (on chromosome Y) was observed. Moreover, in participant LT05, an intact provirus was integrated in a large non-genic region on chromosome 16 in proximity to non-centromeric satellite DNA; this genomic region was previously shown to harbor intact proviruses in two ECs.^9^ Collectively, the integration of intact proviruses in centromeric/peri-centromeric satellite DNA was observed in 6/8 LT-ART study participants; moreover, the proportion of intact proviruses located in centromeric or peri-centromeric satellite/micro-satellite DNA among all independent intact proviruses detected in LT-ART individuals reached 32.26% (Figure 2B). We also detected several additional intact proviruses that were located in non-genic DNA outside of centromeres or satellite/micro-satellite DNA. These proviruses were frequently positioned at a profound distance from host transcriptional start sites (TSSs) (Figure 2C). Overall, the frequencies of intact proviruses in all non-genic locations were comparable between LT-ART individuals and ECs but significantly smaller in ART-treated persons with more limited ART duration (m-ART) (Figures 2B and 2D).

### Integration of intact proviruses in KRAB-ZNF genes

In addition to intact proviruses in centromeric satellite/micro-satellite DNA, we identified several large clones of intact proviruses in Krüppel-associated box zinc-finger protein (KRAB-ZNF) genes, most of which were present in a defined region on chromosome 19. These genes are densely packed with the heterochromatin protein 1 (HP1),^25^ which promotes a chromatin compartment that effectively excludes RNA polymerase from nuclear DNA.^26^ Moreover, ZNF gene bodies are loaded with repressive H3K9me3 histone modifications that bind to HP1 and are deposited by the human silencing hub (HUSH) complex, which has a known role for transcriptional repression of endogenous and exogenous retroviruses.^27^^,^^28^^,^^29^ We observed large clones of intact proviruses integrated in KRAB-ZNF genes in participants LT06, LT07, and LT08; in LT07 (Figures 2A and S3), almost the entire detectable intact proviral reservoir consisted of one clone integrated in a ZNF gene on chromosome 19. In total, 23.08% of all independent intact proviruses detected in LT-ART individuals were integrated in ZNF genes; most of them were located on chromosome 19 (Figure 2B). Notably, 2 of the intact proviruses integrated in ZNF genes (in participants LT06 and LT07) were integrated in regions predicted to be part of lamina-associated domains (LADs), based on DNA adenine methyltransferase identification (DamID) assays conducted previously.^30^ LADs are located at the inner sides of nuclear membranes, represent infrequent targets for HIV-1 integration, and contain mostly transcriptionally silent heterochromatin.^31^ In addition, integration sites of intact proviruses in ZNF genes were positioned in immediate proximity to inhibitory H3K9me3 histone modifications, as determined by alignment to chromatin immunoprecipitation sequencing (ChIP-seq) data from primary CD4 T cells (Figure S4); integration in proximity to H3K9me3-enriched chromatin was associated with resistance to HIV-1 reactivation in *in vitro* assays in our previous work.^8^ Transcriptional repression of HIV-1 proviruses integrated in ZNF genes has also been suggested by other investigators.^32^^,^^33^ However, transcriptional behavior of intact proviruses in ZNF genes may be influenced by the precise proviral positioning relative to epigenetic chromatin features, and not all intact proviruses in ZNF genes may be in a state of "deep latency".

Only a relatively small number of intact proviruses (19.35% of all independent proviruses) in LT-ART individuals were located in active transcription units other than ZNF genes. Such integration sites were almost exclusively observed for intact proviruses from one single study participant (LT06). For a systematic analysis of integration sites from LT-ART individuals, we aligned integration site coordinates of intact proviruses to genome-wide maps of host gene transcription (determined by RNA-seq in primary CD4 T cell subsets) and of 3D chromatin interactions (determined by Hi-C) from reference datasets.^16^^,^^24^ These studies demonstrated an enrichment of intact proviruses from LT-ART individuals in marked distance to host TSS (Figure 2C) and in the 3D heterochromatin compartments B2 and B4 (Figure 2D). Overall, our results indicate that the proviral integration site profile in LT-ART individuals is characterized by disproportionate frequencies of intact proviruses integrated in heterochromatin locations and closely resembles the integration site landscape in ECs.^9^

### Transcriptional activity of intact proviruses

**Figure 3:**
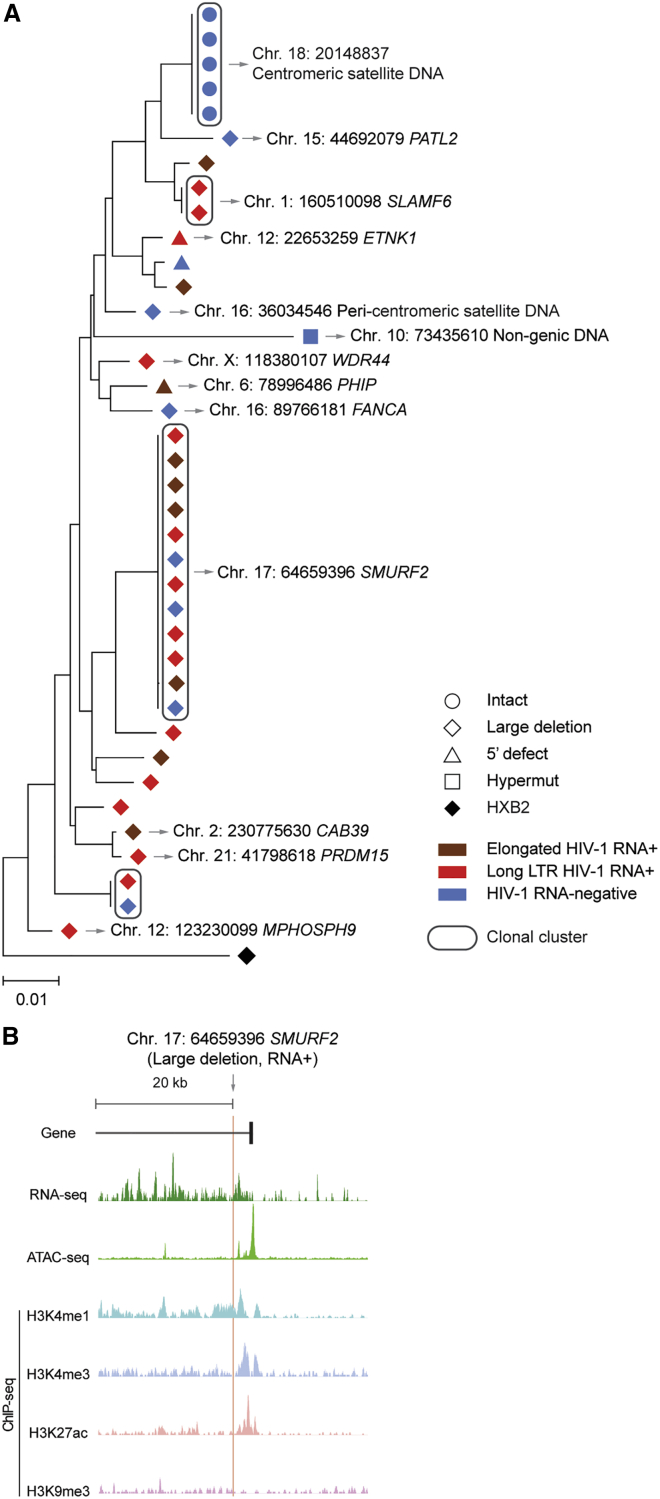
Transcriptional activity of single HIV-1 proviruses from study participant LT03 (A) Maximum-likelihood phylogenetic tree of individual proviruses isolated from LT03 using PRIP-seq. Chromosomal integration sites are indicated where available; genes harboring the integration site are shown where applicable. Color coding reflects the transcriptional activity of proviral species. (B) Genome browser snapshot indicating RNA-seq, Assay for Transposase-Accessible Chromatin using sequencing (ATAC-seq), and ChIP-seq reads surrounding the chromosomal integration site of the indicated proviral sequence from study participant LT03; RNA-seq and ATAC-seq reads are derived from reference data of HIV-1-infected ART-treated persons and were first described in Einkauf et al.^16^; ChIP-seq reads from the reference dataset of the ROADMAP consortium^34^ were used.

To further evaluate whether proviruses in centromeric satellite/micro-satellite locations are indeed transcriptionally repressed and in a state of deep latency, we performed parallel assessments of HIV-1 RNA, integration sites and proviral sequences (PRIP-seq) from single HIV-1-infected cells, using a previously described protocol^8^; such a direct *ex vivo* analysis can evaluate proviral transcriptional activity in response to physiological reactivation signals that occur *in vivo*. These experiments demonstrated that the large genome-intact proviral clone integrated in centromeric satellite DNA of chromosome 18 in LT03 was completely transcriptionally silent across all 5 member sequences; an additional defective proviral sequence integrated in centromeric satellite DNA on chromosome 16 was also transcriptionally silent (Figure 3A). In contrast, multiple other defective clonal and non-clonal proviral sequences with integration sites in genic chromosomal locations were detected; most of these defective proviruses transcribed HIV-1 long-LTR and/or more elongated HIV-1 RNA transcripts (Figure 3A). In particular, we noted one large defective proviral clone that was integrated in the SMURF2 gene in immediate proximity to activating epigenetic chromatin features and displayed high-level proviral transcriptional activity (Figures 3A and 3B). PRIP-seq assays with additional proviruses integrated in satellite DNA (n = 34), described in our previous work,^8^ also suggested effective transcriptional repression conferred by chromosomal integration into such distinct chromatin locations. As an additional analysis step, we performed traditional quantitative viral outgrowth assays (qVOAs) that evaluate infectious viral particle production after *in vitro* stimulation with non-physiological reactivation agents and high-dose exogenous cytokine support, using an assay protocol that, in our previous work, readily permitted detection of replication-competent viruses in persons with shorter durations (<2.3–13 years) of ART^12^^,^^35^ and in ECs.^9^ A combined calculated estimate of n = 307 genome-intact proviruses from a combined total of 15.7 million isolated CD4 T cells from 5 study participants (LT02, LT03, LT06, LT07, and LT08; Table S3) was tested; viral outgrowth was observed for none of these proviruses after 14 days of *in vitro* culture and for n = 2 proviruses (from study participants LT02 and LT03) after 21 days of culture (Figure 2A). These results, combined with data from *in vitro* models of viral latency,^22^^,^^23^ support our hypothesis that HIV-1 proviruses integrated in satellite DNA or in heterochromatin regions are in a state of deep latency and difficult to reactivate. However, we explicitly do not claim that such proviruses are completely resistant against any type of viral reactivation signals and have reached a state of irreversible latency.

### Longitudinal selection of intact proviruses in deep latency

**Figure 4:**
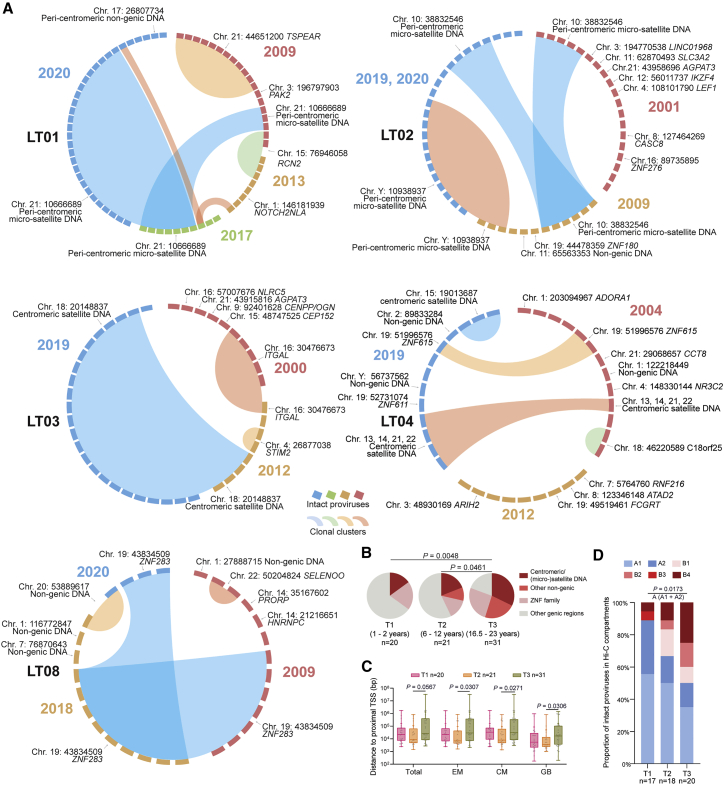
Longitudinal evolution of intact proviral reservoir landscape during long-term antiretroviral therapy (A) CIRCOS plots reflecting the chromosomal locations of intact proviruses at indicated time points in five study participants (LT01–LT04, LT08). Each symbol reflects one intact provirus. Clonal sequences, defined by identical integration sites and/or complete sequence identity, are highlighted. (B) Pie charts reflecting longitudinal evolution of chromosomal integration site landscapes of intact proviruses at indicated time points. Data indicate proportions of intact proviruses in indicated chromosomal locations. (C) Chromosomal distance between integration sites of intact proviruses to most proximal host TSS, as determined by RNA-seq in CD4 T cells from reference datasets in total, effector-memory (EM), or central-memory (CM) primary CD4 T cells or from genome browser (GB).^16^ Box and Whisker plots show median, interquartile ranges and minimum/maximum. (D) Proportions of genome-intact proviral sequences in structural compartments/subcompartments A and B, as determined by Hi-C seq data.^24^ Integration sites not covered in the reference dataset were excluded. For T3, data from 2017 and 2020 from LT01 were pooled. (B–D) Clonal sequences are counted once. n reflects the number of integration sites. p values were calculated by chi-square tests in (B) and (D), and by FDR-adjusted two-sided Kruskal-Wallis nonparametric tests in (C), adjusted for multiple comparison testing where applicable.

To better evaluate the mechanisms leading to the specific reservoir profile in LT-ART individuals, we conducted a longitudinal analysis of integration sites for intact proviruses in a subgroup of n = 5 participants from whom PBMC samples collected at prior time points were available (Figures 4A and S5; Tables S1 and S2). We noted that intact proviruses after relatively short-term ART (T1) were frequently located in introns of highly expressed genes, which represent the preferred target sites for HIV-1 integrase. After moderate time periods of ART (T2), intact proviruses were again mostly integrated in genic locations; proviruses in centromeric satellite/micro-satellite DNA were detectable at slightly increased frequencies at this time relative to the earlier analysis time point (Figure 4B). Although the majority of intact proviruses during earlier stages of treatment were detected as singlets, larger clones of intact proviruses were noted after moderate durations of ART; these proviral clones were frequently integrated in genes associated with or involved in regulation of cell proliferation, such as Reticulocalbin 2 (RCN2) (participant LT01), Thrombospondin Type Laminin G Domain And EAR Repeats (TSPEAR) (participant LT01), or Integrin Subunit Alpha L (ITGAL) (participant LT03). At the final time point (T3), the integration site landscape of intact proviruses was strongly biased toward heterochromatin regions, as described above. Together, these longitudinal evaluations suggested a progressively increasing chromosomal distance of intact proviruses to host TSS, coupled with an enrichment for intact proviruses integrated in 3D chromatin compartments with heterochromatin features (Figures 4C and 4D). Importantly, intact proviruses detected in centromeric satellite DNA and, to a lesser extent, in ZNF genes detected at early treatment time points, frequently persisted long term and were detectable at the final time point of follow-up. In contrast, proviruses in genic positions tended to disappear over time and were rarely detected at multiple time points. Together, our work supports the notion that intact proviruses in heterochromatin positions have a selection advantage that permits long-term persistence over two decades of ART. In contrast, intact proviruses in more permissive chromatin seem to be actively selected against.

**Figure 5:**
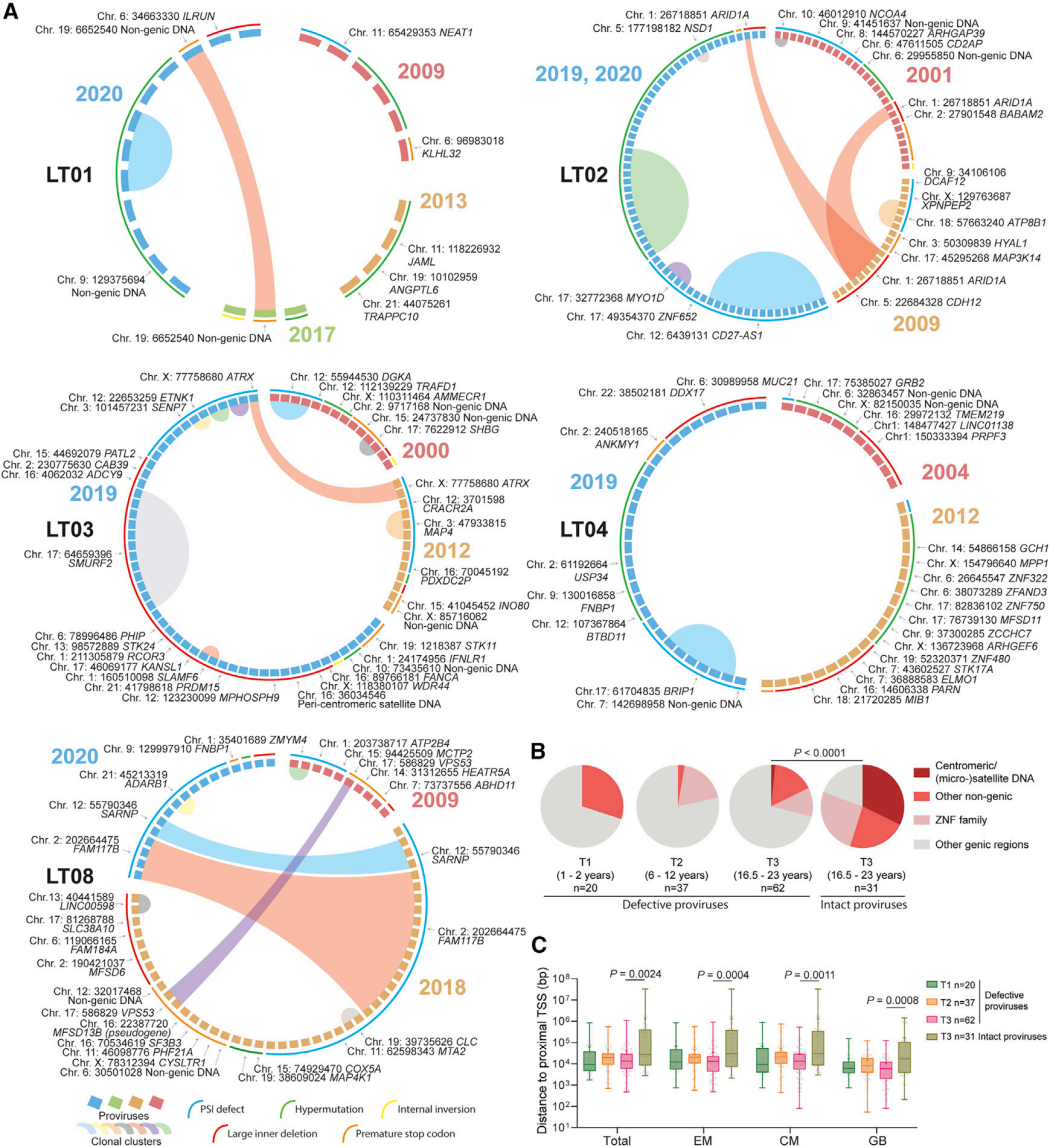
Proviral landscape of defective HIV-1 proviruses in long-term ART-treated individuals (A) CIRCOS plots reflecting the chromosomal locations of defective proviruses at indicated time points in five study participants (LT01–LT04, LT08). Each symbol reflects one defective provirus. Clonal sequences, defined by integration sites and/or complete sequence identity, are highlighted. Color-coded arches around the plots indicate types of defects in HIV-1 genomes. (B) Proportions of intact and defective proviruses with indicated integration site features at time points T1–T3. (C) Chromosomal distance between integration sites of intact and defective proviruses to most proximal TSS, as determined by RNA-seq in CD4 T cells from reference datasets^16^ at indicated time points. Box and Whisker plots show median, interquartile ranges and minimum/maximum. (B and C) Data from defective proviruses after 20 years of suppressive antiretroviral therapy are cross-sectionally compared with corresponding data from intact proviruses. A complete list of defective proviruses and their corresponding chromosomal location is indicated in Table S1. p values were calculated by a chi-square test in (B) and by an FDR-adjusted two-sided Kruskal-Wallis nonparametric test in (C), adjusted for multiple comparison testing where applicable. n reflects the number of integration sites.

For comparative purposes, we also conducted a longitudinal analysis of the proviral integration site profile for defective proviruses in the same subgroup of our study participants for whom intact proviruses had been longitudinally analyzed (n = 5). Generally, we observed that the proviral integration site landscape of defective proviruses differed profoundly from intact proviruses; specifically, defective proviruses were predominantly integrated in genic regions and there was only one single defective provirus that was located in satellite DNA (in LT03) (Figures 5A and 5B). These data are consistent with previously described preferences for HIV-1 integration site locations.^36^^,^^37^^,^^38^^,^^39^ In addition, the chromosomal distance between host TSS and defective proviruses remained relatively stable over time, in apparent contrast to intact proviruses (Figure 5C). Together, these results demonstrate striking differences in the longitudinal evolution of intact versus defective proviruses. Such differences suggest that immune selection mechanisms may differentially affect intact and defective proviruses, possibly because host selection mechanisms may be able to distinguish intact proviruses that are able to produce fully functional viral particles from defective proviruses that, depending on the type of defect, may only produce HIV-1 RNA and proteins. A distinct trajectory of intact and defective proviruses during ART is also suggested by prior studies that focused on a quantitative longitudinal analysis of proviral frequencies during ART.^40^^,^^41^^,^^42^^,^^43^

### Integration site profile of persons with drug-free control of HIV after treatment interruption

**Figure 6:**
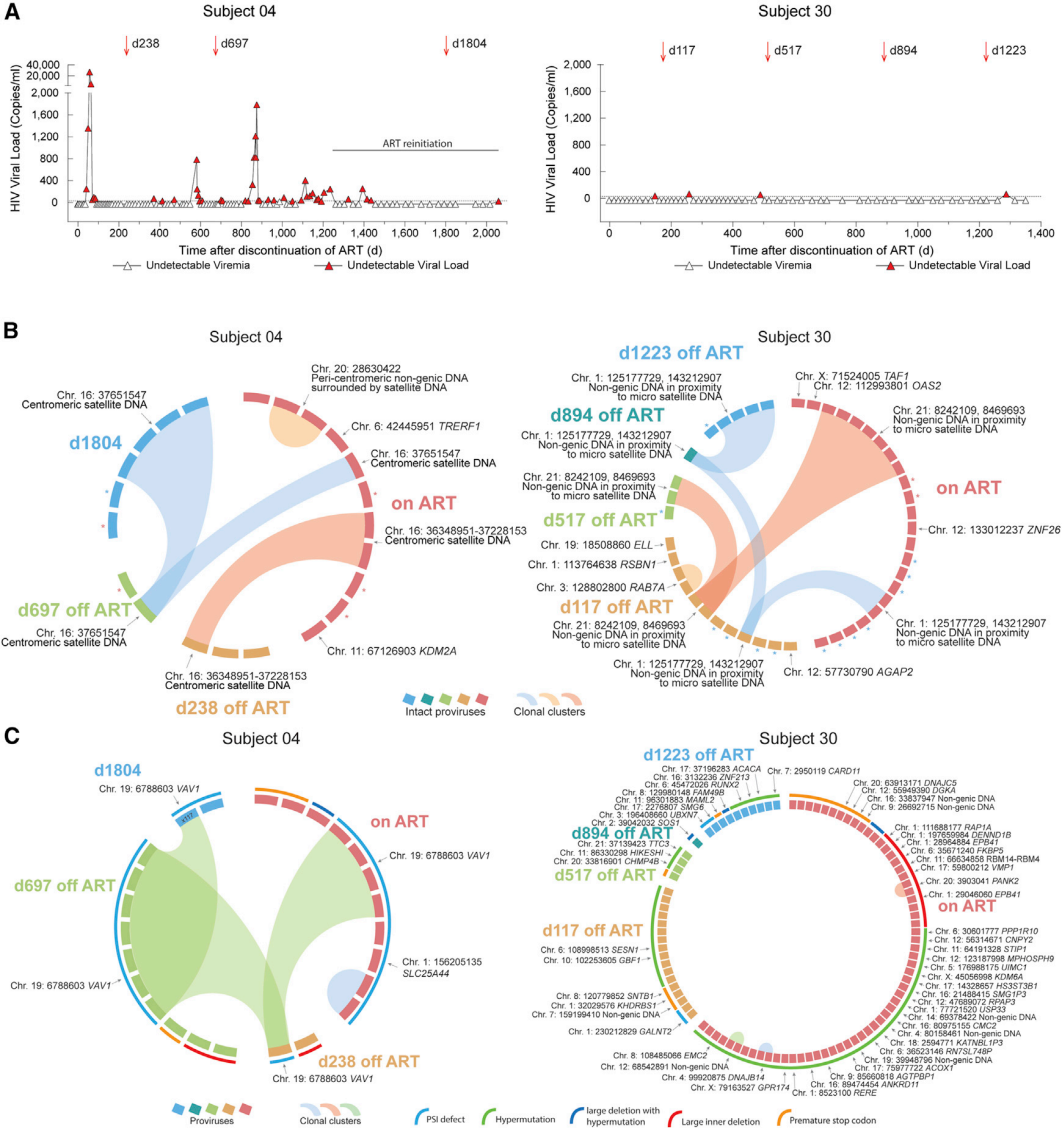
Longitudinal dynamics of intact proviruses in two individuals with post-treatment control (A) Longitudinal analysis of HIV-1 plasma viral load in study persons 04 and 30. PBMC sampling time points are indicated by arrows. Day 0 is the first day of treatment interruption. (B) CIRCOS plots indicating longitudinal evolution of intact proviruses and their corresponding chromosomal integration sites. Each symbol reflects one intact provirus. Clonal sequences, defined by identical integration sites and/or complete sequence identity, are highlighted. In study person 30, two clones were detected in repetitive genomic regions in immediate proximity to micro-satellite DNA; due to the repetitive nature of these regions, the exact chromosomal region could not be definitively identified. ^∗^Intact proviral sequences analyzed without identification of integration sites that differ by 1 or 2 base pairs from adjacent clonal sequences and might be part of the respective clones. (C) CIRCOS plots indicating longitudinal evolution and chromosomal locations of defective HIV-1 proviruses in study persons 04 and 30. Color-coded arches around the plots indicate types of defects in HIV-1 genomes.

Remarkably, the integration site profile in LT-ART individuals closely resembles the block and lock viral reservoir configuration in persons with natural viral control,^9^ suggesting that PLHIV may, over prolonged periods of antiretroviral treatment, mount a level of selection pressure against viral reservoir cells that reduces their ability to effectively fuel rebound viremia in case of treatment interruptions. Consistent with this notion, longer antiretroviral treatment durations were associated with a higher probability for post-treatment, drug-free control in one prior study involving individuals identified during primary infection.^44^ We believe that this question deserves future investigation in dedicated clinical studies that will selectively evaluate the effects of ART discontinuation in PLHIV displaying signs of a block and lock profile for intact proviruses. To address this here, we used MIP-seq to longitudinally profile the integration site landscape of intact proviruses in two persons who had served as placebo recipients in a prior therapeutic vaccination study^45^ and developed drug-free control of HIV-1 after discontinuation of ART^46^ (Figures 6A and S6). These persons resembled post-treatment controllers (PTCs) described in a previous study.^47^ Both study persons had received ART for approximately 6.6 years prior to stopping suppressive antiviral therapy. In study person 04, we observed three different clones of intact proviruses at baseline (immediately prior to stopping ART), all of which were integrated in or surrounded by centromeric satellite DNA; additionally, two intact proviruses in genic locations were also detected at this time (Figure 6B; Table S1). Several spikes of plasma viremia were noted after treatment interruption; however, these spikes were self-limited and did not lead to prolonged viremia. Spikes of plasma viremia became less obvious during the subsequent follow-up period, and the person decided to self-initiate ART at day 1,262 after the initial treatment discontinuation. Over time, a progressive longitudinal accumulation of intact proviruses in centromeric satellite DNA was noticed, while intact proviruses in genic locations were no longer detectable, likely as a result of continuous host selection mechanisms during treatment interruption (Figure 6B). A large genome-defective proviral clone integrated in the Vav Guanine Nucleotide Exchange Factor 1 (VAV1) proto-oncogene was noted at multiple time points in this individual; proviral integration in this gene can lead to insertional mutagenesis supporting cell-autonomous clonal T cell proliferation^48^ (Figure 6C). In study person 30, we observed two clones of intact proviruses integrated in non-genic chromosomal regions in immediate proximity to micro-satellite DNA at baseline; additionally, smaller numbers of intact proviruses in typical genic chromatin locations were also present before treatment interruption (Figure 6B; Table S1). Spikes of plasma viremia were smaller after ART discontinuation in this person (in the setting of less frequent viral load testing compared with study person 04); however, an accumulation of intact proviruses in heterochromatin regions, paired with a de-selection of intact proviruses in genic locations, was also noted in this person. A large number of defective proviruses were identified in person 30, with the vast majority of these proviruses located in typical genic chromosomal locations (Figure 6C).

**Figure 7:**
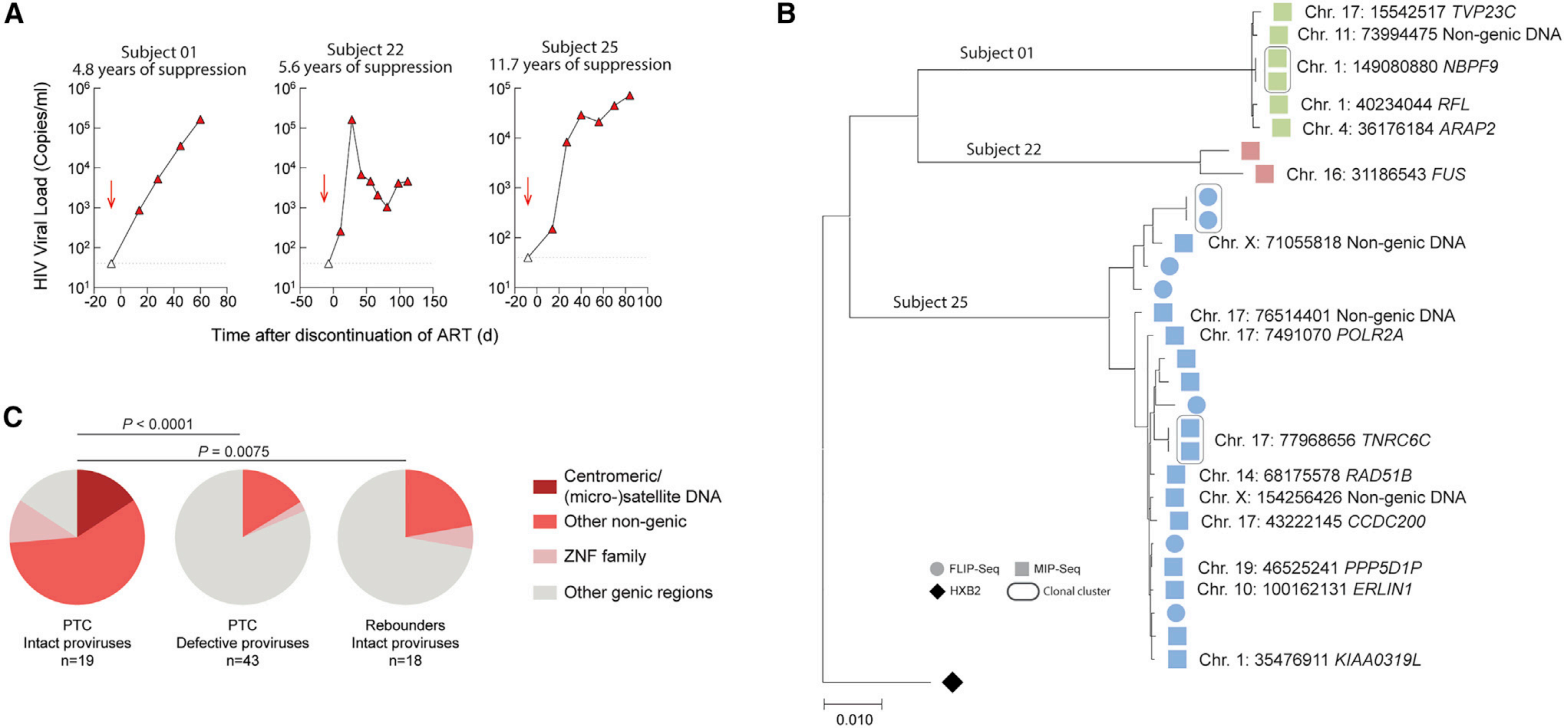
Integration site profile of intact proviruses from study persons with virological rebound after ART interruption (A) Longitudinal analysis of HIV-1 plasma viral load in three study persons who developed rapid viral rebound following ART interruption. PBMC sampling time points are indicated by arrows. (B) Maximum likelihood phylogenetic tree for intact proviruses isolated from PBMC samples prior to ART interruption in three rebounders shown in (A). Chromosomal integration sites are indicated when available. (C) Proportions of intact and defective proviruses with indicated integration sites in PTC. Data from intact proviruses identified in rebounders are shown for comparison. Data from the time point immediately prior to treatment interruption are shown; clonal sequences are counted individually. Significance was calculated using a chi-square test, adjusted for multiple comparison testing. n reflects the number of integration sites.

Using the same analytic approach, we analyzed the integration site profile of intact proviruses in three participants from the same study^45^ who rebounded to high-level viremia following treatment interruption. In these individuals, the majority of intact proviruses detected prior to treatment interruption were integrated in genic locations (Figures 7A and 7B), and no integration of intact proviruses within or close to centromeres, micro-, or macro-satellite DNA was observed, resulting in a markedly different integration site profile of intact proviruses in PTC and rebounders (Figure 7C). Together, these observations suggest that (1) a viral reservoir landscape enriched for intact proviruses integrated in heterochromatin locations has a weaker ability to fuel sustained viral rebound after ART interruption and that (2) PTCs show evidence for accelerated selection of intact proviruses in heterochromatin, likely as a result of stronger antiviral selection forces and, possibly, higher levels of proviral transcriptional reactivation of intact proviruses in euchromatin during off-treatment periods. Such transcriptional reactivation may expose the reservoir cells to antiviral immune effects.

## Discussion

Previous data suggested that it may require 70 or more years of continuous ART to induce a clinically significant decay of HIV-1 reservoir cells;^49^ however, these calculations are based on theoretical extrapolations of experimental results from *in vitro* outgrowth assays, which may not capture the true biological behavior of HIV-1 reservoir cells *in vivo*. The growing number of PLHIV who started treatment approximately 20 years ago when triple-drug combination antiretroviral treatment became first available makes it possible to empirically test the evolution and persistence of HIV-1 during two decades of continuous suppressive therapy. Our data suggest that over such extended periods of time, intact proviruses integrated in permissive chromatin are more likely to be recognized and eliminated by innate or adaptive immune effector cells, or by cell-intrinsic immune recognition pathways that result in cell death.^50^ In contrast, the small subset of intact proviruses integrated in repressive chromatin locations seems to have a longitudinal selection advantage and persists long-term; integration in such repressive chromosomal segments might protect against immune-mediated elimination and can be regarded as a biomarker of selection pressure that viral reservoir cells have been exposed to *in vivo*. These selection effects seem particularly visible during the second decade of suppressive ART, during which the reservoir profile appears to undergo a marked transformation. Notably, evidence of selection of proviruses with specific chromosomal integration site features was also noted in other retroviral diseases, specifically in the context of Human T-lymphotropic virus type 1 (HTLV-1) infection;^51^ however, proviral selection may also be influenced by cell-intrinsic host features of virally infected cells that are independent of the chromosomal integration site, as recently suggested by studies involving single-cell transcriptional profiling of HIV-1 reservoir cells.^52^^,^^53^

The integration site profiles of intact proviruses shown here in LT-ART-treated persons closely resemble those of ECs,^9^ raising the possibility that once reservoir cells show footprints of intense immune selection pressure, an attenuated viral reservoir profile with a weaker ability to drive sustained rebound viremia may have been reached. In support of that, our work demonstrated that the transcriptional activity of proviruses integrated in satellite DNA and heterochromatin regions, when assessed directly *ex vivo* by the PRIP-seq assay, is profoundly reduced relative to proviruses in alternative locations. Furthermore, *in vitro* outgrowth assays with cells from LT-ART persons demonstrated poor susceptibility of proviruses integrated in heterochromatin locations, although repetitive rounds of *in vitro* stimulation may have more effectively reactivated viral replication.^39^ However, it remains uncertain to what degree *in vitro* viral outgrowth assays can serve as an appropriate model for *in vivo* viral outgrowth, as host immune factors—including virus-specific T and B cells—are typically not included in such assays and results from *in vitro* viral outgrowth assays do not correlate with kinetics of viral rebound during antiretroviral treatment interruptions. Moreover, the responsiveness of proviruses to reactivation signals is likely to differ profoundly between artificial *in vitro* culture conditions and physiological *in vivo* systems. In fact, prior studies have shown that proviral outgrowth in *in vitro* assays, typically involving stimulation with Phytohaemagglutinin (PHA) and high-dose IL-2, can follow a stochastic pattern,^39^^,^^54^^,^^55^ while *in vivo* viral outgrowth may be more prominently influenced by epigenetic features at the proviral integration site. Therefore, restriction of proviral outgrowth *in vivo* that is mediated by the proviral chromosomal location and the surrounding epigenetic microenvironment may not be appropriately captured by *in vitro* viral outgrowth assays.

Whether integrations site profiles of intact proviruses do influence the propensity for viral outgrowth *in vivo* will, in our opinion, require future prospective clinical studies in which treatment is selectively interrupted in persons in whom a blocked and locked integration site pattern is noted. In this manuscript, we provided a retrospective analysis of integration site locations in persons with post-treatment control during a prior study.^46^ Owing to the small number of such persons that have been described in the literature, this analysis was limited to two study persons; however, within these individuals, integration of intact proviruses in repressive heterochromatin was already noted prior to treatment interruption and progressively increased after treatment cessation. In contrast, a more ordinary chromosomal integration site landscape with predominance of intact proviruses integrated in genes was noted in three persons with rapid viral rebound during treatment interruption. Although larger studies will be necessary to further investigate this in the future, our work suggests that it might not only be the presence or absence of antiviral immune responses that determines drug-free control of HIV-1. Rather, we argue that the degree of selection that has occurred in viral reservoir cells may be a distinguishing feature of persons who maintain drug-free undetectable viremia, with or without prior ART. Future technologies designed to evaluate integration sites of much larger numbers of intact proviruses will be informative to determine whether enrichment in heterochromatin locations is also detectable in subdominant intact proviral species that cannot be efficiently captured with currently available experimental approaches but may contribute to viral rebound. Additionally, it will be critical to investigate whether the progressive selection of proviruses in heterochromatin during LT-ART is associated with specific immune mechanisms. Indeed, recent work suggests a role for CD8 T cells in influencing viral transcriptional behavior^56^ and an accelerated decline of intact proviruses after treatment with broadly neutralizing antibodies.^57^ Collectively, our work reveals strong evidence that HIV-1 reservoir cells are subject to host selection mechanisms that can, over extended periods of ART, profoundly transform the viral reservoir landscape. Whether such selection mechanisms truly act in favor of the host and translate into clinically significant benefits remains to be determined in future clinical studies.

## STAR★Methods

### Key resources table

**Table:** 

REAGENT or RESOURCE	SOURCE	IDENTIFIER
**Biological samples**		

PBMC samples from study participants living with HIV	This study, Massachusetts General Hospital, University of California at San Francisco, Case Western Reserve University, National Institutes of Health (NIH) Clinical Center	N/A

**Chemicals, peptides, and recombinant proteins**		

Buffer RLT Plus	Qiagen	Cat#1053393
Invitrogen Dynabeads MyOne Streptavidin C1	ThermoFisher Scientific	Cat#65002
Invitrogen SUPERase⋅In RNase Inhibitor	ThermoFisher Scientific	Cat#AM2694
Invitrogen dNTP mix (10mM each)	ThermoFisher Scientific	Cat#18427088
AMPure XP beads	Beckman Coulter	Cat#A63882
5M NaCl	ThermoFisher Scientific	Cat#AM9760G
10M NaOH	Millipore Sigma	Cat#72068
0.5M EDTA (pH 8.0)	Promega	Cat#V4231
UltraPure 1M Tris-HCI Buffer (pH 7.5)	ThermoFisher Scientific	Cat#15567027
1M MgCl_2_	ThermoFisher Scientific	Cat#AM9530G
2M KCl	ThermoFisher Scientific	Cat#AM9640G
1M DTT	Millipore Sigma	Cat#646563
TWEEN 20 (50% Solution)	ThermoFisher Scientific	Cat#003005
Betaine solution (5M)	Millipore Sigma	Cat#B0300
BioLegend Cell Activation Cocktail (without Brefeldin A)	BioLegend	Cat#423302
Recombinant IL-2	NIH AIDS Reagent program	www.hivreagentprogram.org
PHA	ThermoFisher Scientific	Cat#R30852701
AZT	NIH AIDS Reagent Program	www.hivreagentprogram.org

**Critical commercial assays**		

DNeasy Blood and Tissue Kit	Qiagen	Cat#69504
ddPCR Supermix for Probes (No dUTP)	Bio-Rad	Cat#1863024
Invitrogen SuperScript II Reverse Transcriptase kit	ThermoFisher Scientific	Cat#18064022
KAPA HiFi HotStart ReadyMix	Roche	Cat#7958935001
REPLI-g Single Cell Kit	Qiagen	Cat#150345
REPLI-g Advanced Single Cell Kit	Qiagen	Cat#150365
Invitrogen Platinum Taq DNA Polymerase High Fidelity	ThermoFisher Scientific	Cat#11304102
Stemcell EasySep Human CD4+ T Cell Isolation Kit	Stemcell Technologies	Cat#17952
Britelite plus Reporter Gene Assay System	PerkinElmer	Cat#6066761
PicoPure RNA Isolation Kit	Applied Biosystems	Cat#0204

**Deposited data**		

Ensembl (v86)	Ensembl	http://oct2016.archive.ensembl.org/index.html
UCSC Genome Browser	UCSC	http://genome.ucsc.edu
GENCODE (v32)	GENCODE	https://www.gencodegenes.org/human/release_32.html
Roadmap database	Roadmap Epigenomics et al.^34^	http://www.roadmapepigenomics.org/
ENCODE database	National Human Genome Research Institute, 2012	https://www.encodeproject.org/
Hi-C data from CD4 T cells	Einkauf et al.^8^	GEO ID: GSE168337
RNA-Seq data from CD4 T cells	Jiang et al.^9^	GEO ID: GSE 144334

**Oligonucleotides**		

See Table S4 for List of Primers/Probes	Einkauf et al.^8^ and Jiang et al.^9^	N/A
Quantitative Synthetic Human immunodeficiency virus 1 (HIV-1) RNA	ATCC	Cat#VR-3245S

**Software and algorithms**		

QuantaSoft software	Bio-Rad	Cat#1864011
Ultracycler v1.0	Seed and Wang, personal communication	https://dnacore.mgh.harvard.edu/new-cgi-bin/site/pages/viral_genome_sequencing_pages/viral_genome_sequencing_data.jsp
Automated in-house proviral intactness bioinformatic pipeline in Python	Lee et al.^12^	https://github.com/BWH-Lichterfeld-Lab/Intactness-Pipeline
Los Alamos National Laboratory (LANL) HIV Sequence Database Hypermut 2.0	Rose and Korber^58^	https://www.hiv.lanl.gov/content/sequence/HYPERMUT/background.html
MUSCLE	Edgar^59^	http://www.drive5.com/muscle/
Geneious Prime 2021.0.3	Biomatters	https://www.geneious.com/download/
bwa-mem	Li and Durbin^60^	http://maq.sourceforge.net/
RepeatMasker	Institute for Systems Biology	http://www.repeatmasker.org/
STAR aligner software	ENCODE, version 2.5.1b	https://www.encodeproject.org/software/star/
Prism	Graphpad, version 8.2.1	https://www.graphpad.com/scientific-software/prism
R	R Core Team and R Foundation for Statistical Computing, version 3.5.3	https://www.r-project.org
FastQC	Babraham Bioinformatics, version 0.11.	https://www.bioinformatics.babraham.ac.uk
Samtools	Genome Research Limited, version 1.14	http://www.htslib.org
MACS2	MACS3 project team, version 2.1.1.20160309	https://github.com/macs3-project/MACS
Recombinant Identification Program	Los Alamos National Laboratory	https://www.hiv.lanl.gov/content/sequence/RIP/RIP.html
Bowtie2	Langmead and Salzberg,^61^ version 2.2.9	http://bowtie-bio.sourceforge.net/bowtie2/index.shtml
Python	Python Software Foundation, version 3.9	https://www.python.org/
Omixon HLA Explore	Omixon, version beta	https://www.omixon.com/products/hla-explore/
Biorender	Biorender	https://biorender.com

**Other**		

QX200 Droplet Digital PCR System	Bio-Rad	https://www.bio-rad.com/en-us/life-science/digital-pcr/qx200-droplet-digital-pcr-system
C1000 Touch Thermal Cycler with 96-Well Fast Reaction Module	Bio-Rad	Cat#1851196
Quantify One and ChemiDoc MP Image Lab	Bio-Rad	https://www.bio-rad.com/en-us/product/chemidoc-mp-imaging-system
ThermoMixer C	Eppendorf	Cat#5382000023
96-Well PCR Post Magnet Low Elution Plate	Permagen	Cat#LE400
DynaMag-96 Side Skirted Magnet	ThermoFisher Scientific	Cat#12027
DynaMag-2 Magnet	ThermoFisher Scientific	Cat#12321D
Illumina MiSeq performed by MGH CCIB DNA Core facility	Illumina/MGH CCIB DNA Core	https://dnacore.mgh.harvard.edu/new-cgi-bin/site/pages/index.jsp
Fluidigm Access Array	Fluidigm	https://www.standardbio.com/products-services/instruments/access-array
NextSeq 500 Instrument	Illumina	https://www.illumina.com/systems/sequencing-platforms/nextseq.html

### Resource availability

#### Lead contact

Further information and requests for resources and reagents should be directed to and will be fulfilled by the lead contact, Mathias Lichterfeld (mlichterfeld@partners.org).

#### Materials availability

This study did not generate new unique reagents.

### Experimental model and subject details

#### Study participants

HIV-1-infected study participants were recruited at the Massachusetts General Hospital (MGH), the Brigham and Women’s Hospital (BWH, both in Boston, MA, USA), the University of California, San Francisco (UCSF) at the Zuckerberg San Francisco General Hospital (San Francisco, CA, USA) and the NIH Clinical Center (Bethesda, MD, USA). Samples were also collected at Case Western Reserve University (Cleveland, OH, USA) under protocol A5321 of the AIDS Clinical Trials Group (ACTG). The influence of gender was not measured in this study. PBMC were obtained according to protocols approved by the respective Institutional Review Boards. Clinical characteristics of study participants are summarized in Figures 6, 7, and S1. Numbers of cells assayed are shown in Table S2.

#### Study approval

Study participants gave written informed consent to participate in accordance with the Declaration of Helsinki. The study was approved by the institutional review boards of MGH, BWH, Case Western Reserve University, NIH and UCSF.

### Method details

#### Droplet digital PCR

PBMC or CD4+ cells were subjected to DNA extraction using commercial kits (Qiagen DNeasy #69504). Total HIV-1 DNA was amplified with ddPCR Supermix for Probes (no dUTPs, Bio-Rad), using primers (final concentration 1μM) and probes (final concentration 350nM) described previously^12^ (127 bp 5′LTR-gag amplicon; HXB2 coordinates 684–810). Droplets were prepared using the Automated Droplet Generator (Bio-Rad) and PCR was performed using the following program: 95°C for 10 min, 45 cycles of 94°C for 30s and 60°C for 1 min, 98°C for 10 min. The droplets were subsequently read by the QX200 droplet reader and data were analyzed using QuantaSoft software (Bio-Rad).

#### HLA class I typing

HLA typing was performed using a targeted next-generation sequencing method, as described previously.^62^ Briefly, locus-specific primers were used to amplify polymorphic exons of HLA-A, B, C (exons 1–4) genes with Fluidigm Access Array (Fluidigm). The Fluidigm PCR amplicons were pooled and subjected to sequencing on an Illumina MiSeq sequencer (Illumina, San Diego, CA). HLA alleles and genotypes were called using the Omixon HLA Explore software (Omixon).

#### Whole genome amplification

Extracted DNA was diluted to single viral genome levels according to ddPCR results, so that 1 provirus was present in approximately 20-30% of wells. Subsequently, DNA in each well was subjected to multiple displacement amplification (MDA) with phi29 polymerase (Qiagen REPLI-g Single Cell Kit #150345), per the manufacturer’s protocol. Following this unbiased whole genome amplification,^63^ DNA from each well was split and separately subjected to near full-length viral sequencing and integration site analysis, as described below. If necessary, a second-round MDA reaction was performed to increase the amount of available DNA.

#### HIV near full-genome sequencing

DNA resulting from whole-genome amplification reactions was subjected to HIV-1 near full-genome amplification using a 1-amplicon and/or non-multiplexed 5-amplicon approach, as described before.^16^ PCR products were visualized by agarose gel electrophoresis (Quantify One and ChemiDoc MP Image Lab, BioRad). All near full-length and/or 5-amplicon positive amplicons were subjected to Illumina MiSeq sequencing at the MGH DNA Core facility. Resulting short reads were *de novo* assembled using Ultracycler v1.0 and aligned to HXB2 to identify large deleterious deletions (<8000bp of the amplicon aligned to HXB2), out-of-frame indels, premature/lethal stop codons, internal inversions, or packaging signal defects (≥15 bp insertions and/or deletions relative to HXB2), using an automated in-house pipeline written in Python programming language (https://github.com/BWH-Lichterfeld-Lab/Intactness-Pipeline),^64^ consistent with prior studies.^12^^,^^14^^,^^18^ Presence/absence of apolipoprotein B mRNA editing enzyme catalytic polypeptide-like (APOBEC) 3G/3F-associated hypermutations was determined using the Los Alamos National Laboratory (LANL) HIV Sequence Database Hypermut 2.0^58^ program. Viral sequences that lacked all mutations listed above were classified as “genome-intact” sequences. Sequence alignments were performed using MUSCLE.^59^ Phylogenetic distances between sequences were examined using maximum likelihood trees in MEGA (www.megasoftware.net) and MAFFT (https://mafft.cbrc.jp/alignment/software), and visualized using Highlighter plots (https://www.hiv.lanl.gov/content/sequence/HIGHLIGHT/highlighter_top.html). Viral sequences were considered clonal if they had completely identical consensus sequences; single nucleotide variations in primer binding sites were not considered for clonality analysis. Clades of intact HIV-1 proviral sequences were determined using the LANL HIV Sequence Database Recombinant Identification Program.^65^ Within intact HIV-1 clade B sequences, the proportions of optimal CTL epitopes (restricted by autologous HLA class I alleles) matching the clade B consensus sequence and CTL escape variants restricted by selected HLA class I alleles and supertypes described in the LANL HIV Immunology Database (www.hiv.lanl.gov) were determined. The number of sequence mutations associated with HLA class I-mediated pressure were calculated in clade-B proviruses as previously described.^13^ The sensitivity of proviral species to broadly-neutralizing antibodies (bnAb) was estimated as previously described.^66^ Briefly, full-length viral sequences were aligned to the reference HIV-1 clade-B sequence HXB2 and the HIV-1 *env* gene sequence was identified using Gene Cutter (https://www.hiv.lanl.gov/content/sequence/GENE_CUTTER/cutter.html). The number of amino acid signature sites that are associated with sensitivity to four bnAb classes (V2, V3, CD4 binding site, and membrane proximal external region) within the env sequence from each HIV-1 provirus was calculated according to data described previously.^66^

#### Integration site analysis

Integration sites associated with each viral sequence were obtained using integration site loop amplification (ISLA), using a protocol previously described by Wagner et al.^37^; DNA produced by whole-genome amplification was used as template. For selected clonal sequences, viral-host junction regions were also amplified using primers annealing upstream of the integration site in host DNA and downstream of the integration site in viral DNA. Resulting PCR products were subjected to next-generation sequencing using Illumina MiSeq. MiSeq paired-end FASTQ files were demultiplexed; small reads (142 bp) were then aligned simultaneously to human reference genome GRCh38 and HIV-1 reference genome HXB2 using bwa-mem.^60^ Biocomputational identification of integration sites was performed according to previously-described procedures^37^^,^^67^: Briefly, chimeric reads containing both human and HIV-1 sequences were evaluated for mapping quality based on (i) HIV-1 coordinates mapping to the terminal nucleotides of the viral genome, (ii) absolute counts of chimeric reads, (iii) depth of sequencing coverage in the host genome adjacent to the viral integration site. The final list of integration sites and corresponding chromosomal annotations was obtained using Ensembl (v86, www.ensembl.org), the UCSC Genome Browser (www.genome.ucsc.edu) and GENCODE (v39, www.gencodegenes.org). Repetitive genomic sequences harboring HIV-1 integration sites were identified using RepeatMasker (www.repeatmasker.org).

#### PRIP-seq

Parallel analysis of HIV-1 RNA, integration sites and proviral sequences from single HIV-1-infected cells was performed according to the protocol described previously.^8^ Briefly, PBMCs were diluted to single HIV-1-infected cells according to ddPCR results, so that one virally-infected cell was present in approximately 20-30% of wells, and lysed with Buffer RLT Plus (Qiagen). After annealing with modified biotinylated HIV-1-specific primers, viral RNA was magnetically separated from genomic DNA according to the G&T-Seq protocol and subjected to reverse transcription with SuperScript II Reverse Transcriptase (Invitrogen) as described previously.^8^ Amplified cDNA was subjected to absolute quantification using ddPCR with primers and probes targeting different regions of HIV-1 RNA, as previously described.^5^ Isolated gDNA was subjected to whole genome amplification, HIV-1 near full-genome sequencing, and integration site analysis as described in method details.

#### RNA-seq, ChIP-seq, Hi-C

RNA-Seq data generated from primary total and memory CD4+ T cells and described in a previous publication^9^ were used for analysis. These data are deposited in NCBI GEO: GSE144334. ChIP-Seq data derived from primary human memory CD4+ T cells and included in the ROADMAP consortium portal (http://www.roadmapepigenomics.org/) were used for analysis. Hi-C Seq data used in this study were described by Rao et al.^24^

#### Quantitative viral outgrowth assays

Viral outgrowth assays were performed as described in our previous work.^9^^,^^12^^,^^35^ CD4^+^ cells were isolated from PBMCs using the EasySep Human CD4 Positive Selection Kit II (STEMCELL Technologies 17852). Cells were plated in limiting dilutions on 96-well plates based on the intact provirus reservoir size determined through FLIP-Seq, so that a single provirus was present in approximately 20% of wells. ACH2 cells were run as positive controls on each plate. Irradiated feeder PBMCs were added at 1 × 10^5^ cells per well. Cells were activated with 1 μg/ml PHA for 3 days, which was subsequently washed away and 10,000 MOLT-4 CCR5^+^ cells (NIH AIDS Reagent Program, 4984) per well were added to propagate infection. On the 14th and 21th days, culture supernatants from each well were individually incubated with 10,000 TZM-bl cells (NIH AIDS Reagent Program, 8129) to drive *Tat*-dependent luciferase production. On the 16th and 23rd days, TZM-bl cells were lysed, and luciferase activity was measured using Britelite Plus (PerkinElmer, 6066761). Luciferase-positive wells were defined as having signal levels that were >5-fold higher than negative controls. Cells from positive wells were then collected and plated into bottom compartments of Transwell tissue-culture inserts (Costar 6.5 mm Transwells, 0.4-μm pore polyester membrane inserts, STEMCELL, 38024), while 1 × 10^6^ MOLT-4 cells were placed in top compartments. After five additional days of culture, MOLT-4 cells from the upper wells were collected and subjected to FLIP-Seq for identification of intact proviruses.

### Quantification and statistical analysis

#### Statistics

Data are presented as pie charts, bar charts, scatter plots with individual values or Box and Whisker plots (indicating the median, minimum, maximum and interquartile ranges). Differences were tested for statistical significance using Mann Whitney U tests, Fishers’ exact tests, chi-square tests, and two-sided Kruskal-Wallis nonparametric tests, as appropriate. *P* < 0.05 was considered significant; false discovery rate (FDR) correction was performed using the Benjamini-Hochberg method.^68^ Statistical details can be found in the corresponding figure legends. Analyses were performed using GraphPad Prism and R software.

## Data Availability

• RNA-Seq data was deposited to Gene Expression Omnibus (GEO) with the following accession numbers GEO: GSE144334, which can also be found in the key resources table. • Proviral integration sites and their transcriptional activity are listed in Table S1. • Proviral sequences: Due to study participant confidentiality concerns, viral sequencing data cannot be publicly released, but will be made available to investigators upon reasonable request and after signing a data transfer agreement. • This paper does not report original code. Publicly available software and code used in this study are listed in the key resources table. • Any additional information required to reanalyze the data reported in this paper is available from the lead contact upon request.

## Supplemental information

Document S1. Figures S1–S6, Tables S2–S4, and supplemental references

Table S1. Integration sites of all intact and defective proviruses in all study participants, related to Figure 2

Document S2. Article plus supplemental information

## References

[1] Finzi D., Hermankova M., Pierson T., Carruth L.M., Buck C., Chaisson R.E., Quinn T.C., Chadwick K., Margolick J., Brookmeyer R. (1997). Identification of a reservoir for HIV-1 in patients on highly active antiretroviral therapy. Science.

[2] Wong J.K., Hezareh M., Günthard H.F., Havlir D.V., Ignacio C.C., Spina C.A., Richman D.D. (1997). Recovery of replication-competent HIV despite prolonged suppression of plasma viremia. Science.

[3] Chun T.W., Carruth L., Finzi D., Shen X., DiGiuseppe J.A., Taylor H., Hermankova M., Chadwick K., Margolick J., Quinn T.C. (1997). Quantification of latent tissue reservoirs and total body viral load in HIV-1 infection. Nature.

[4] Ruelas D.S., Greene W.C. (2013). An integrated overview of HIV-1 latency. Cell.

[5] Yukl S.A., Kaiser P., Kim P., Telwatte S., Joshi S.K., Vu M., Lampiris H., Wong J.K. (2018). HIV latency in isolated patient CD4(+) T cells may be due to blocks in HIV transcriptional elongation, completion, and splicing. Sci. Transl. Med..

[6] Pollack R.A., Jones R.B., Pertea M., Bruner K.M., Martin A.R., Thomas A.S., Capoferri A.A., Beg S.A., Huang S.H., Karandish S. (2017). Defective HIV-1 proviruses are expressed and can be recognized by cytotoxic T lymphocytes, which shape the proviral landscape. Cell Host Microbe.

[7] Imamichi H., Smith M., Adelsberger J.W., Izumi T., Scrimieri F., Sherman B.T., Rehm C.A., Imamichi T., Pau A., Catalfamo M. (2020). Defective HIV-1 proviruses produce viral proteins. Proc. Natl. Acad. Sci. USA.

[8] Einkauf K.B., Osborn M.R., Gao C., Sun W., Sun X., Lian X., Parsons E.M., Gladkov G.T., Seiger K.W., Blackmer J.E. (2022). Parallel analysis of transcription, integration, and sequence of single HIV-1 proviruses. Cell.

[9] Jiang C., Lian X., Gao C., Sun X., Einkauf K.B., Chevalier J.M., Chen S.M.Y., Hua S., Rhee B., Chang K. (2020). Distinct viral reservoirs in individuals with spontaneous control of HIV-1. Nature.

[10] Elsheikh M.M., Tang Y., Li D., Jiang G. (2019). Deep latency: A new insight into a functional HIV cure. EBioMedicine.

[11] Lian X., Gao C., Sun X., Jiang C., Einkauf K.B., Seiger K.W., Chevalier J.M., Yuki Y., Martin M., Hoh R. (2021). Signatures of immune selection in intact and defective proviruses distinguish HIV-1 elite controllers. Sci. Transl. Med..

[12] Lee G.Q., Orlova-Fink N., Einkauf K., Chowdhury F.Z., Sun X., Harrington S., Kuo H.H., Hua S., Chen H.R., Ouyang Z. (2017). Clonal expansion of genome-intact HIV-1 in functionally polarized Th1 CD4+ T cells. J. Clin. Invest..

[13] Carlson J.M., Brumme C.J., Martin E., Listgarten J., Brockman M.A., Le A.Q., Chui C.K., Cotton L.A., Knapp D.J., Riddler S.A. (2012). Correlates of protective cellular immunity revealed by analysis of population-level immune escape pathways in HIV-1. J. Virol..

[14] Pinzone M.R., VanBelzen D.J., Weissman S., Bertuccio M.P., Cannon L., Venanzi-Rullo E., Migueles S., Jones R.B., Mota T., Joseph S.B. (2019). Longitudinal HIV sequencing reveals reservoir expression leading to decay which is obscured by clonal expansion. Nat. Commun..

[15] Bui J.K., Sobolewski M.D., Keele B.F., Spindler J., Musick A., Wiegand A., Luke B.T., Shao W., Hughes S.H., Coffin J.M. (2017). Proviruses with identical sequences comprise a large fraction of the replication-competent HIV reservoir. PLoS Pathog..

[16] Einkauf K.B., Lee G.Q., Gao C., Sharaf R., Sun X., Hua S., Chen S.M., Jiang C., Lian X., Chowdhury F.Z. (2019). Intact HIV-1 proviruses accumulate at distinct chromosomal positions during prolonged antiretroviral therapy. J. Clin. Invest..

[17] Hosmane N.N., Kwon K.J., Bruner K.M., Capoferri A.A., Beg S., Rosenbloom D.I., Keele B.F., Ho Y.C., Siliciano J.D., Siliciano R.F. (2017). Proliferation of latently infected CD4(+) T cells carrying replication-competent HIV-1: potential role in latent reservoir dynamics. J. Exp. Med..

[18] Hiener B., Horsburgh B.A., Eden J.S., Barton K., Schlub T.E., Lee E., von Stockenstrom S., Odevall L., Milush J.M., Liegler T. (2017). Identification of genetically intact HIV-1 proviruses in specific CD4(+) T cells from effectively treated participants. Cell Rep..

[19] Cho A., Gaebler C., Olveira T., Ramos V., Saad M., Lorenzi J.C.C., Gazumyan A., Moir S., Caskey M., Chun T.W., Nussenzweig M.C. (2022). Longitudinal clonal dynamics of HIV-1 latent reservoirs measured by combination quadruplex polymerase chain reaction and sequencing. Proc. Natl. Acad. Sci. USA.

[20] Carteau S., Hoffmann C., Bushman F. (1998). Chromosome structure and human immunodeficiency virus type 1 cDNA integration: centromeric alphoid repeats are a disfavored target. J. Virol..

[21] Schröder A.R., Shinn P., Chen H., Berry C., Ecker J.R., Bushman F. (2002). HIV-1 integration in the human genome favors active genes and local hotspots. Cell.

[22] Lewinski M.K., Bisgrove D., Shinn P., Chen H., Hoffmann C., Hannenhalli S., Verdin E., Berry C.C., Ecker J.R., Bushman F.D. (2005). Genome-wide analysis of chromosomal features repressing human immunodeficiency virus transcription. J. Virol..

[23] Jordan A., Defechereux P., Verdin E. (2001). The site of HIV-1 integration in the human genome determines basal transcriptional activity and response to Tat transactivation. EMBO J..

[24] Rao S.S., Huntley M.H., Durand N.C., Stamenova E.K., Bochkov I.D., Robinson J.T., Sanborn A.L., Machol I., Omer A.D., Lander E.S., Aiden E.L. (2014). A 3D map of the human genome at kilobase resolution reveals principles of chromatin looping. Cell.

[25] Vogel M.J., Guelen L., de Wit E., Peric-Hupkes D., Lodén M., Talhout W., Feenstra M., Abbas B., Classen A.K., van Steensel B. (2006). Human heterochromatin proteins form large domains containing KRAB-ZNF genes. Genome Res..

[26] Strom A.R., Emelyanov A.V., Mir M., Fyodorov D.V., Darzacq X., Karpen G.H. (2017). Phase separation drives heterochromatin domain formation. Nature.

[27] Douse C.H., Tchasovnikarova I.A., Timms R.T., Protasio A.V., Seczynska M., Prigozhin D.M., Albecka A., Wagstaff J., Williamson J.C., Freund S.M.V. (2020). TASOR is a pseudo-PARP that directs HUSH complex assembly and epigenetic transposon control. Nat. Commun..

[28] Tchasovnikarova I.A., Timms R.T., Matheson N.J., Wals K., Antrobus R., Göttgens B., Dougan G., Dawson M.A., Lehner P.J. (2015). Gene silencing. Epigenetic silencing by the HUSH complex mediates position-effect variegation in human cells. Science.

[29] Matkovic R., Morel M., Lanciano S., Larrous P., Martin B., Bejjani F., Vauthier V., Hansen M.M.K., Emiliani S., Cristofari G. (2022). TASOR epigenetic repressor cooperates with a CNOT1 RNA degradation pathway to repress HIV. Nat. Commun..

[30] Robson M.I., de Las Heras J.I., Czapiewski R., Sivakumar A., Kerr A.R.W., Schirmer E.C. (2017). Constrained release of lamina-associated enhancers and genes from the nuclear envelope during T-cell activation facilitates their association in chromosome compartments. Genome Res..

[31] Marini B., Kertesz-Farkas A., Ali H., Lucic B., Lisek K., Manganaro L., Pongor S., Luzzati R., Recchia A., Mavilio F. (2015). Nuclear architecture dictates HIV-1 integration site selection. Nature.

[32] Huang A.S., Ramos V., Oliveira T.Y., Gaebler C., Jankovic M., Nussenzweig M.C., Cohn L.B. (2021). Integration features of intact latent HIV-1 in CD4+ T cell clones contribute to viral persistence. J. Exp. Med..

[33] Cole B., Lambrechts L., Gantner P., Noppe Y., Bonine N., Witkowski W., Chen L., Palmer S., Mullins J.I., Chomont N. (2021). In-depth single-cell analysis of translation-competent HIV-1 reservoirs identifies cellular sources of plasma viremia. Nat. Commun..

[34] Kundaje A., Meuleman W., Ernst J., Bilenky M., Yen A., Heravi-Moussavi A., Kheradpour P., Zhang Z., Wang J., Roadmap Epigenomics Consortium (2015). Integrative analysis of 111 reference human epigenomes. Nature.

[35] Buzon M.J., Sun H., Li C., Shaw A., Seiss K., Ouyang Z., Martin-Gayo E., Leng J., Henrich T.J., Li J.Z. (2014). HIV-1 persistence in CD4 T cells with stem cell-like properties. Nat. Med..

[36] Maldarelli F., Wu X., Su L., Simonetti F.R., Shao W., Hill S., Spindler J., Ferris A.L., Mellors J.W., Kearney M.F. (2014). HIV latency. Specific HIV integration sites are linked to clonal expansion and persistence of infected cells. Science.

[37] Wagner T.A., McLaughlin S., Garg K., Cheung C.Y., Larsen B.B., Styrchak S., Huang H.C., Edlefsen P.T., Mullins J.I., Frenkel L.M. (2014). HIV latency. Proliferation of cells with HIV integrated into cancer genes contributes to persistent infection. Science.

[38] Cohn L.B., Silva I.T., Oliveira T.Y., Rosales R.A., Parrish E.H., Learn G.H., Hahn B.H., Czartoski J.L., McElrath M.J., Lehmann C. (2015). HIV-1 integration landscape during latent and active infection. Cell.

[39] Ho Y.C., Shan L., Hosmane N.N., Wang J., Laskey S.B., Rosenbloom D.I., Lai J., Blankson J.N., Siliciano J.D., Siliciano R.F. (2013). Replication-competent noninduced proviruses in the latent reservoir increase barrier to HIV-1 cure. Cell.

[40] Peluso M.J., Bacchetti P., Ritter K.D., Beg S., Lai J., Martin J.N., Hunt P.W., Henrich T.J., Siliciano J.D., Siliciano R.F. (2020). Differential decay of intact and defective proviral DNA in HIV-1-infected individuals on suppressive antiretroviral therapy. JCI Insight.

[41] Falcinelli S.D., Kilpatrick K.W., Read J., Murtagh R., Allard B., Ghofrani S., Kirchherr J., James K.S., Stuelke E., Baker C. (2020). Longitudinal dynamics of intact HIV proviral DNA and outgrowth virus frequencies in a cohort of ART-treated individuals. J. Infect. Dis..

[42] Simonetti F.R., White J.A., Tumiotto C., Ritter K.D., Cai M., Gandhi R.T., Deeks S.G., Howell B.J., Montaner L.J., Blankson J.N. (2020). Intact proviral DNA assay analysis of large cohorts of people with HIV provides a benchmark for the frequency and composition of persistent proviral DNA. Proc. Natl. Acad. Sci. USA.

[43] Gandhi R.T., Cyktor J.C., Bosch R.J., Mar H., Laird G.M., Martin A., Collier A.C., Riddler S.A., Macatangay B.J., Rinaldo C.R. (2021). Selective decay of intact HIV-1 proviral DNA on antiretroviral therapy. J. Infect. Dis..

[44] Fidler S., Olson A.D., Bucher H.C., Fox J., Thornhill J., Morrison C., Muga R., Phillips A., Frater J., Porter K. (2017). Virological blips and predictors of post treatment viral control after stopping ART started in primary HIV infection. J. Acquir. Immune Defic. Syndr..

[45] Sneller M.C., Justement J.S., Gittens K.R., Petrone M.E., Clarridge K.E., Proschan M.A., Kwan R., Shi V., Blazkova J., Refsland E.W. (2017). A randomized controlled safety/efficacy trial of therapeutic vaccination in HIV-infected individuals who initiated antiretroviral therapy early in infection. Sci. Transl. Med..

[46] Blazkova J., Gao F., Marichannegowda M.H., Justement J.S., Shi V., Whitehead E.J., Schneck R.F., Huiting E.D., Gittens K., Cottrell M. (2021). Distinct mechanisms of long-term virologic control in two HIV-infected individuals after treatment interruption of anti-retroviral therapy. Nat. Med..

[47] Sáez-Cirión A., Bacchus C., Hocqueloux L., Avettand-Fenoel V., Girault I., Lecuroux C., Potard V., Versmisse P., Melard A., Prazuck T. (2013). Post-treatment HIV-1 controllers with a long-term virological remission after the interruption of early initiated antiretroviral therapy ANRS VISCONTI Study. PLoS Pathog..

[48] Liu R., Yeh Y.J., Varabyou A., Collora J.A., Sherrill-Mix S., Talbot C.C., Mehta S., Albrecht K., Hao H., Zhang H. (2020). Single-cell transcriptional landscapes reveal HIV-1-driven aberrant host gene transcription as a potential therapeutic target. Sci. Transl. Med..

[49] Siliciano J.D., Kajdas J., Finzi D., Quinn T.C., Chadwick K., Margolick J.B., Kovacs C., Gange S.J., Siliciano R.F. (2003). Long-term follow-up studies confirm the stability of the latent reservoir for HIV-1 in resting CD4+ T cells. Nat. Med..

[50] Li P., Kaiser P., Lampiris H.W., Kim P., Yukl S.A., Havlir D.V., Greene W.C., Wong J.K. (2016). Stimulating the RIG-I pathway to kill cells in the latent HIV reservoir following viral reactivation. Nat. Med..

[51] Melamed A., Fitzgerald T.W., Wang Y., Ma J., Birney E., Bangham C.R.M. (2022). Selective clonal persistence of human retroviruses in vivo: radial chromatin organization, integration site, and host transcription. Sci. Adv..

[52] Collora J.A., Liu R., Pinto-Santini D., Ravindra N., Ganoza C., Lama J.R., Alfaro R., Chiarella J., Spudich S., Mounzer K. (2022). Single-cell multiomics reveals persistence of HIV-1 in expanded cytotoxic T cell clones. Immunity.

[53] Weymar G.H.J., Bar-On Y., Oliveira T.Y., Gaebler C., Ramos V., Hartweger H., Breton G., Caskey M., Cohn L.B., Jankovic M., Nussenzweig M.C. (2022). Distinct gene expression by expanded clones of quiescent memory CD4(+) T cells harboring intact latent HIV-1 proviruses. Cell Rep..

[54] Hansen M.M.K., Martin B., Weinberger L.S. (2019). HIV latency: stochastic across multiple scales. Cell Host Microbe.

[55] Hataye J.M., Casazza J.P., Best K., Liang C.J., Immonen T.T., Ambrozak D.R., Darko S., Henry A.R., Laboune F., Maldarelli F. (2019). Principles Governing Establishment versus Collapse of HIV-1 cellular Spread. Cell Host Microbe.

[56] McBrien J.B., Mavigner M., Franchitti L., Smith S.A., White E., Tharp G.K., Walum H., Busman-Sahay K., Aguilera-Sandoval C.R., Thayer W.O. (2020). Robust and persistent reactivation of SIV and HIV by N-803 and depletion of CD8(+) cells. Nature.

[57] Gaebler C., Nogueira L., Stoffel E., Oliveira T.Y., Breton G., Millard K.G., Turroja M., Butler A., Ramos V., Seaman M.S. (2022). Prolonged viral suppression with anti-HIV-1 antibody therapy. Nature.

[58] Rose P.P., Korber B.T. (2000). Detecting hypermutations in viral sequences with an emphasis on G --> A hypermutation. Bioinformatics.

[59] Edgar R.C. (2004). MUSCLE: multiple sequence alignment with high accuracy and high throughput. Nucleic Acids Res..

[60] Li H., Durbin R. (2009). Fast and accurate short read alignment with Burrows-Wheeler transform. Bioinformatics.

[61] Langmead B., Salzberg S.L. (2012). Fast gapped-read alignment with Bowtie 2. Nat. Methods.

[62] Digitale J.C., Callaway P.C., Martin M., Nelson G., Viard M., Rek J., Arinaitwe E., Dorsey G., Kamya M., Carrington M. (2021). HLA Alleles B^∗^53:01 and C^∗^06:02 Are Associated With Higher Risk of P. falciparum Parasitemia in a Cohort in Uganda. HLA alleles B(^∗^)53. Front. Immunol..

[63] Burtt N.P. (2011). Whole-genome amplification using Phi29 DNA polymerase. Cold Spring Harb. Protoc..

[64] Lee G.Q., Reddy K., Einkauf K.B., Gounder K., Chevalier J.M., Dong K.L., Walker B.D., Yu X.G., Ndung'u T., Lichterfeld M. (2019). HIV-1 DNA sequence diversity and evolution during acute subtype C infection. Nat. Commun..

[65] Siepel A.C., Halpern A.L., Macken C., Korber B.T. (1995). A computer program designed to screen rapidly for HIV type 1 intersubtype recombinant sequences. AIDS Res. Hum. Retrovir..

[66] Bricault C.A., Yusim K., Seaman M.S., Yoon H., Theiler J., Giorgi E.E., Wagh K., Theiler M., Hraber P., Macke J.P. (2019). HIV-1 neutralizing antibody signatures and application to epitope-targeted vaccine design. Cell Host Microbe.

[67] Serrao E., Cherepanov P., Engelman A.N. (2016). Amplification, next-generation sequencing, and genomic DNA mapping of retroviral integration sites. J. Vis. Exp..

[68] Benjamini Y.H., Yosef (1995). Controlling the false discovery rate: A practical and powerful approach to multiple testing. J. R. Stat. Soc. B Methodol..

