# Supplementary material for: Progressive transformation of the HIV-1 reservoir cell profile over two decades of antiviral therapy
Source: Cell Host Microbe. 2023 Jan 11;31(1):83–96.e5. doi: 10.1016/j.chom.2022.12.002 (PMC9839361; doi:10.1016/j.chom.2022.12.002)
Supplement: Document S1. Figures S1–S6, Tables S2–S4, and supplemental references [file mmc1.pdf]

Cell Host & Microbe, Volume *31*

Supplemental information

Progressive transformation of the HIV-1 reservoir cell profile over two decades of antiviral therapy

Xiaodong Lian, Kyra W. Seiger, Elizabeth M. Parsons, Ce Gao, Weiwei Sun, Gregory T. Gladkov, Isabelle C. Roseto, Kevin B. Einkauf, Matthew R. Osborn, Joshua M. Chevalier, Chenyang Jiang, Jane Blackmer, Mary Carrington, Eric S. Rosenberg, Michael M. Lederman, Deborah K. McMahon, Ronald J. Bosch, Jeffrey M. Jacobson, Rajesh T. Gandhi, Michael J. Peluso, Tae-Wook Chun, Steven G. Deeks, Xu G. Yu, and Mathias Lichterfeld

Supplemental Materials for

Progressive transformation of the HIV-1 reservoir cell profile over two decades of antiviral therapy

Xiaodong Lian, Kyra W. Seiger, Elizabeth M. Parsons, Ce Gao, Weiwei Sun, Gregory T. Gladkov, Isabelle C. Roseto, Kevin B. Einkauf, Matthew R. Osborn, Joshua M. Chevalier, Chenyang Jiang, Jane Blackmer, Mary Carrington, Eric S. Rosenberg, Michael M. Lederman, Deborah McMahon, Ronald J. Bosch, Jeffrey Jacobson, Rajesh T. Gandhi, Michael Peluso, Tae-Wook Chun, Steven G. Deeks, Xu G. Yu, Mathias Lichterfeld

Figure S1

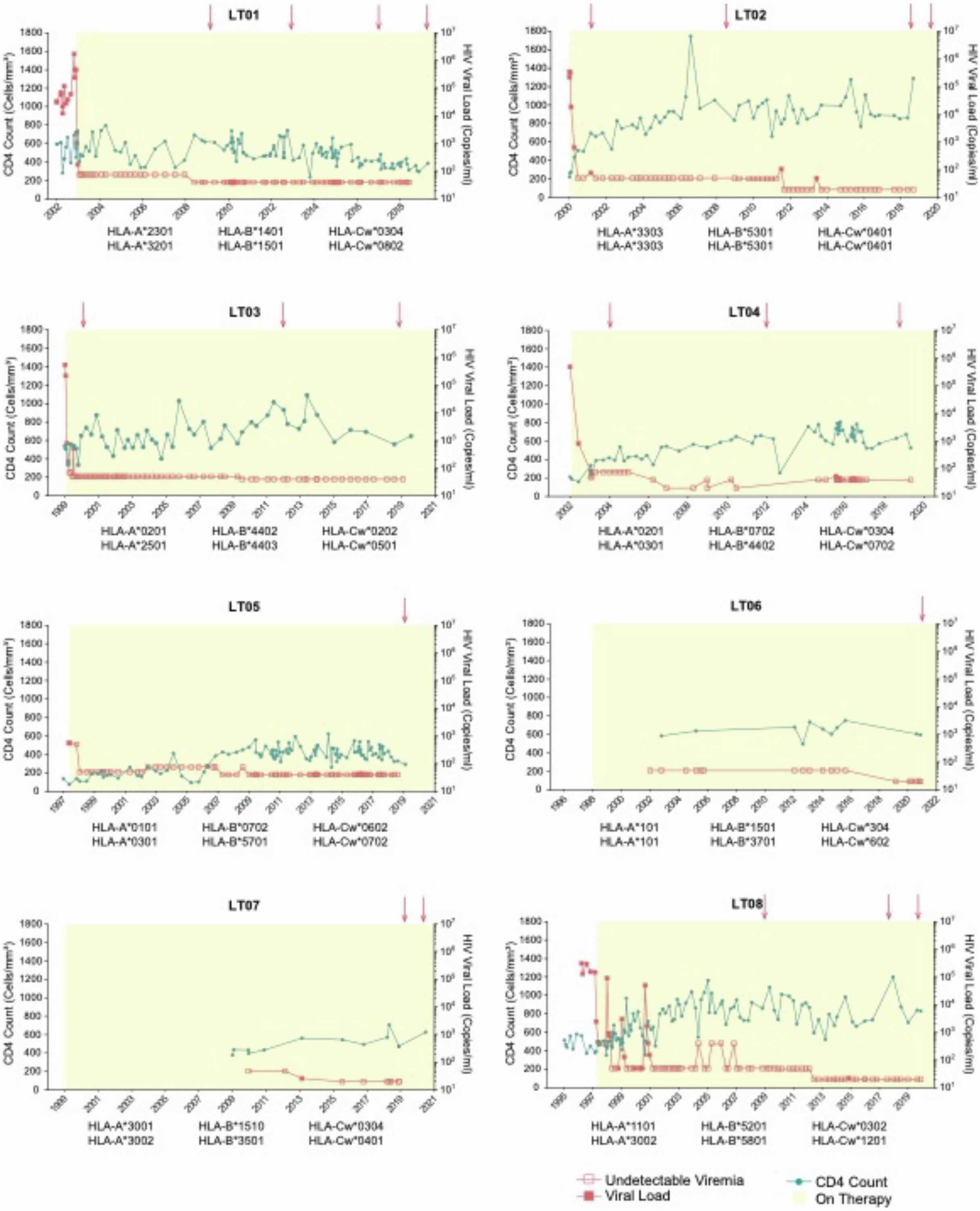

LT01
CD4 Count (Cells/mm³)
HIV Viral Load (Copies/ml)
HLA-A*2301
HLA-A*3201
HLA-B*1401
HLA-B*1501
HLA-Cw*0304
HLA-Cw*0802
LT02
HLA-A*3303
HLA-A*3303
HLA-B*5301
HLA-B*5301
HLA-Cw*0401
HLA-Cw*0401
LT03
HLA-A*0201
HLA-A*2501
HLA-B*4402
HLA-B*4403
HLA-Cw*0202
HLA-Cw*0501
LT04
HLA-A*0201
HLA-A*0301
HLA-B*0702
HLA-B*4402
HLA-Cw*0304
HLA-Cw*0702
LT05
HLA-A*0101
HLA-A*0301
HLA-B*0702
HLA-B*5701
HLA-Cw*0602
HLA-Cw*0702
LT06
HLA-A*101
HLA-A*101
HLA-B*1501
HLA-B*3701
HLA-Cw*304
HLA-Cw*602
LT07
HLA-A*3001
HLA-A*3002
HLA-B*1510
HLA-B*3501
HLA-Cw*0304
HLA-Cw*0401
LT08
HLA-A*1101
HLA-A*3002
HLA-B*5201
HLA-B*5801
HLA-Cw*0302
HLA-Cw*1201
Undetectable Viremia
Viral Load
CD4 Count
On Therapy

Figure S1 (Related to Figure 1): Longitudinal viral loads and CD4 T cell counts in the eight study participants. Timepoints of PBMC sampling are indicated by red arrows. HLA class I alleles are also listed. Duration of ART is indicated by yellow shading. For study participants LT06 and LT07, original viral load and CD4 T cell count data during early stages of ART could not be independently reviewed by study team; however, suppression of viremia during ART was confirmed through provider reports.

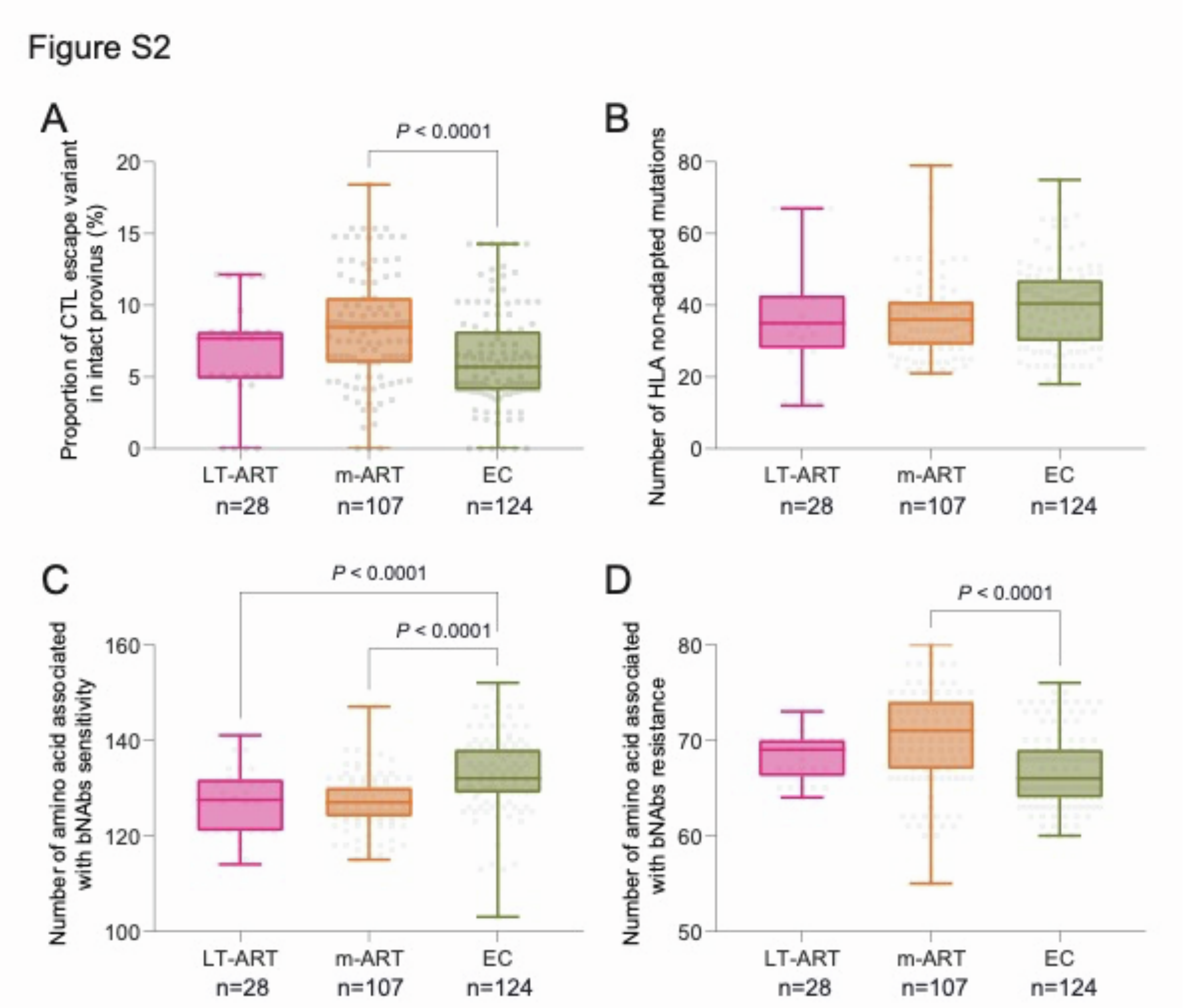

Figure S2 (Related to Figure 1): Immune footprints in intact HIV-1 proviruses. (A): Proportions of clade B CTL epitopes (restricted by autologous HLA class I alleles) within intact proviruses that harbor known escape variants. Defined escape mutations listed in the LANL HIV Immunology Database (www.hiv.lanl.gov) were counted. (B): Numbers of sequence variations without statistically significant associations with autologous HLA class I alleles are shown, determined as described by Carlson et al [S1]. Each dot represents one intact provirus. (C-D): Numbers of amino acid residues associated with sensitivity (C) or resistance (D) to broadly-neutralizing antibodies, calculated as described by Bricault et al [S2]. (A-D): Each symbol represents one intact proviral sequence from indicated study cohort; all intact clade B sequences were included. Clonal sequences are shown once. P-values were calculated using FDR-adjusted two-sided Kruskal-Wallis nonparametric test.

Figure S3

Chr19:43834509:- (*ZNF283*)

5’-TCAAGTAGTGTGTGCCCGTCTGTTGTGTGACTCTGGTAACTAGAGATCCCTCAGACCCTTTTAGTCAGTGTGGAAAATCTCTAGCAGTAATCCACACAATCATTGTAACCATGGCCATACATT
ACATGAACTGTATAATTTCTACATATGCCTATGTCTTGCCTAGAAAAAAAAAGGATGAAATTCCCCAAGTTATGCCATGTATATATTAATGGAATAATACTAATGGAATAATATTAATGAAAT
GATATTAATATATTCATGGCCTAAGTTGAGGAATTTCATCCTTTTTTTTCATTTTTTGAGGGGAGGTTTTATTTATTTTTTTGTTCCCCTTCCATTAATGAGGTTTTGATTCTACTTTTCTTA
TCTTGACTGATGGATTTATTTTGTATCTGAACCTGTCTTAGACATGGTACTCTCCAGATAACTGAATCCAGATAACTGAATCACCTGATCCTATTAGG-3’

Chr19:24126139:- (*ZNF254*)

5’-TCAAGTAGTGTGTGCCCGTCTGTTGTGTGACTCTGGTAACTAGAGATCCCTCAGACCCTTTTAGTCAGTGTGGAAAATCTCTAGCAAATACCACAGGCTCTAATTCTTTCATAGACATAAATG
TAACAAAAACATTCAGACCAAAATACATCTGTGGAAAATTCATAAATGATTTAAGTGTGTGCAGTGTCCCAGGTGAGCACAATGCAAAAAAACACAAAGAAGAAAAAGAAAAGTCTGTTAAAT
TTACTCAATACATCTTTTCCTGTTTCCCACTATACCATAGTGTCTTTACAAGTAAATTGCCAACTCTTGGTTTCTGTTTTAAAG-3’

Chr21:10666689:+ (non-genic peri-centromeric micro-satellite DNA)

5’-TCAAGTAGTGTGTGCCCGTCTGTTGTGTGACTCTGGTAGCTAGAGATCCCTCAGACCCTTTTAGTCAGTGTGGAAAATCTCTAGCAATTCCATTGCATTCCATTCCATTCCATTTGAGACCAT
TCAATTCCATTCCATTCCATTCGAGTCCATTCCATTGCATTCCATTCGATTCCTTTTGAGACCATTCGATTCCATTCCATTTCATTCGAGTCCATTCCATTTGAGTCCATTCCATTGCATTCC
ATTCCATTGTCATCCACTCCATTCTATTCCATTCGAGTCCATTCCATTCCATTAAATTAGAGTCCATTCCATTAAATTCCATTGTATTCCATTTGAGTCCATTCTATTCCATTCCTTTCCATT
CCTGTCCATTCCATTCCACTAGAGTCCATTCCATTCCATTACTTTTGAGTCCATTTCATTCCATTCAATTCTTTTCCAAACAAGTCCATTGGATTCCATTCCATTTCATTCAAGTCCATTCCA
CTCCATTCCATTCTAGTCCGTTACATTCCAGTCCATTTCAATGCAGTCCATTCCATTCTTCTCCATTCAAGTCCATTCCATTCCATTCCATTCGAATCCATTCCATACCATTCCATTCCATTC
CATTCCATTCCATTCCATTCCATTCTTTTCTGTTTGAGCGCACTCTATTCCTTTCGATTCCATTCCATTCCATTGAATTTGAATCCATTCAATTCCGTGTGATTCCATTTGATTCCATTCCAT
TCCATTCCATTAGAGTCCCTTCCATTCAAAGCAATTCCATTCCATTCCATTCCATTCCATTCATCTCCATTCCATACCATTACATTCCGCTTGAGCTCATTCCATTCCATTCCATTCGAGTCC
ATTCCATTGCATTCCATTCCATTCGAGTCCATTGTAGTCCATTCCATTCAAGTCCCTTCCATTGCATTCCATTCCATTCCATTCGAGTCCATTCCGTTCCATTCTATTTCATTTCATTCCATT
CCATTCCATTCCCATG-3’

Chr15:19013687:- (non-genic centromeric satellite DNA)

5’-TCAAGTAGTGTGTGCCCGTCTGTTGTGTGACTCTGGTAACTAGAGATCCCTCAGACCTTTTAGTCAGTGTGGAAAATCTCTAGCATTTCTGAGAATGCTTCTGTCTAGATTTTATATGTAGAT
ATTCCCGTTTCCAACGAAATCCTCAAAGCTATCCAAATATCAACTTGCAGATTCTACAAAAGGAATGTTTCCAAAATGCTGTATCCAAACAAAGGTTCAACTCTGTGAATTGAGGGCATACAT
CACAAAGAAGATTCTGAGAATGCTTCTGTCTAGATTTTATATGAAAATATTCCCGTTTCCAACGAAATCCTCAAAGCTATCCAAATATCCACTTGCAAATGCCACAAAAAGAGTGTTTCCAAA
CTGCTCTGTGAAAAGGAAGGTTCAACTCTGTTAGTTGAGTACACACATCACAAAGAGGTTTCTGAGAATGCTGCTGACTAGTTTTTATTTGAAGATATTTCCCTTTTCACCTTAGGCCTAAGA
GTGCTCGAAATGTCCATTTCCACATACTCCACAAAGTGTGTTTCAAACGTGCTGTATGAAAGGGAATGTTCAACTCTATGAGTTGAATGCAAACATCACAAAGAAGATTCTGAGAATGCTTTT
GTCTAGATTTTATATGAAGATATTCCCGTGTCCAACGAAATTTTCAAAG-3’

Chr2:89833284:+ (non-genic peri-centromeric micro-satellite DNA)

5’-TCAAGTAGTGTGTGCCCGTCTGTTGTGTGACTCTGGTAACTAGAGATCCCTCAGACCTGTTAGTCAGTGTGGAAAATCTCTAGCAGGTTGATTCCATTCCATTGCATTCCATTCCATTCCATT
CCATTCCGTTCCATTCATTCCATTCCATTCCATTCCATTCCATTCCATTCCATTCCATTCCATTCCATTCCATTCCATTCCATTCCACTCNATTCCATTCCATTCCATTCCATTCCACTCCAT
TCCATTCTTGCACTCGGGTTGTTTCCATTCCATTCCATTCCATTCCATTCCATTCCATTCCATTCCATTCCATTCCATTCCATTCCATTCCACTATTCCATTCCATTCCATTCCATAC
CTTTCCA-3’

underlined: 1.U5 binding site red: HIV 3’ LTR sequence blue: human sequence

Figure S3 (Related to Figure 2): Sequences of viral-host junctions of proviruses integrated in satellite/microsatellite DNA or ZNF genes: Sequences of viral-host junctions are shown for selected proviruses from study participants LT01, LT04, LT07, LT08. Sequences of the 1.U5 primer from the ISLA protocol [S3] are underlined.

Figure S4

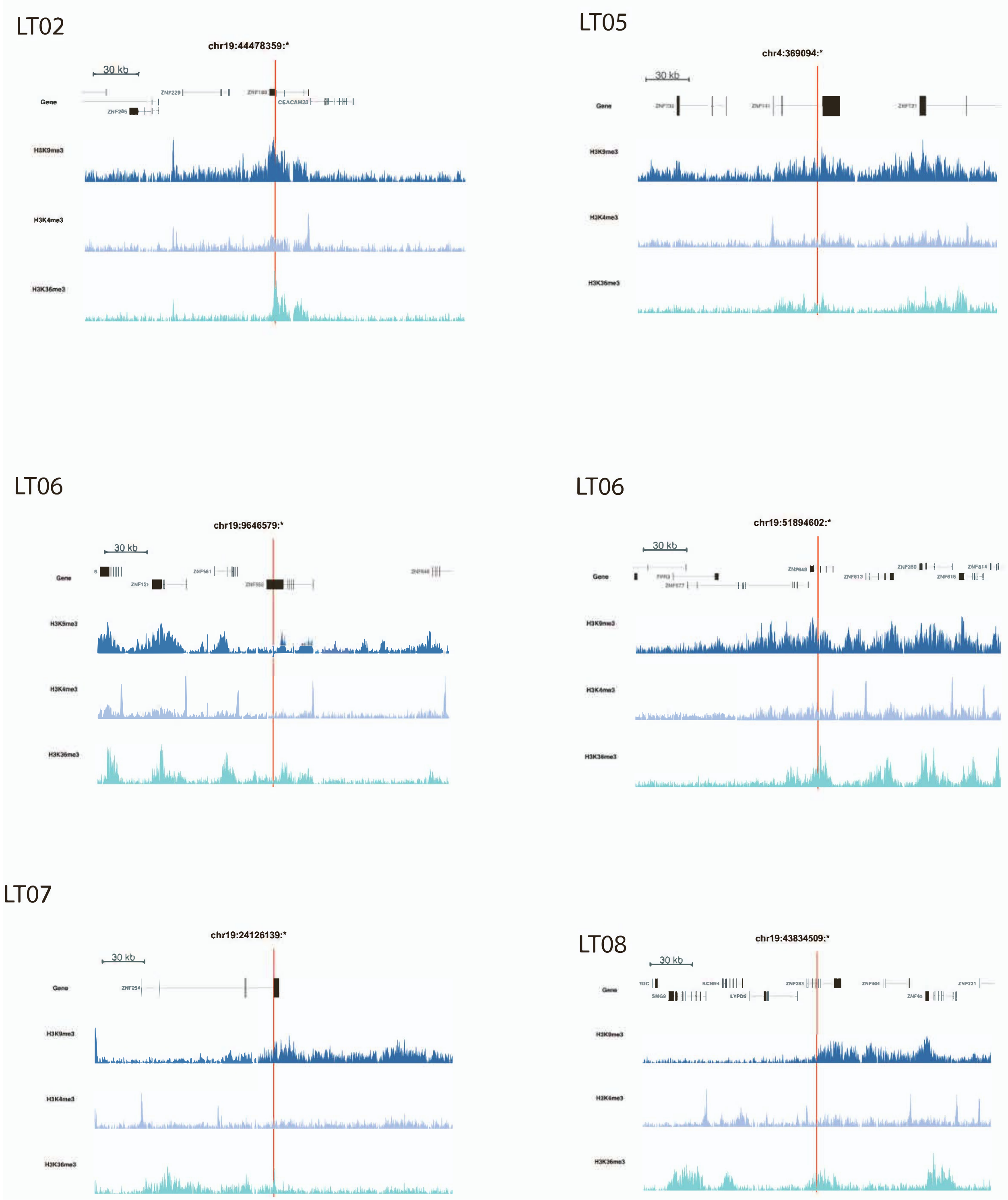

LT02
chr19:44478359:*
30 kb
Gene
CEACAM20
H3K9me3
H3K4me3
H3K36me3
LT05
chr4:369094:*
30 kb
Gene
H3K9me3
H3K4me3
H3K36me3
LT06
chr19:9646579:*
30 kb
Gene
H3K9me3
H3K4me3
H3K36me3
LT06
chr19:51894602:*
30 kb
Gene
H3K9me3
H3K4me3
H3K36me3
LT07
chr19:24126139:*
30 kb
Gene
H3K9me3
H3K4me3
H3K36me3
LT08
chr19:43834509:*
30 kb
Gene
H3K9me3
H3K4me3
H3K36me3

Figure S4 (Related to Figure 2): Chromatin environment of intact proviruses integrated in KRAB-ZNF genes, inferred from reference data. Genome browser shapshots of chromomal integration sites of intact proviruses located in ZNF genes are shown; ChIP-Seq tracks of the inhibitory histone markers H3K9me3, and the activating chromatin mark H3K4me3 from primary CD4 T cells evaulated in the ROADMAP consortium [S4] are included. ChIP-Seq tracks of H3K36me3 (associated with context-depedent transcriptional activation or repression [S5]), are also shown.

Figure S5

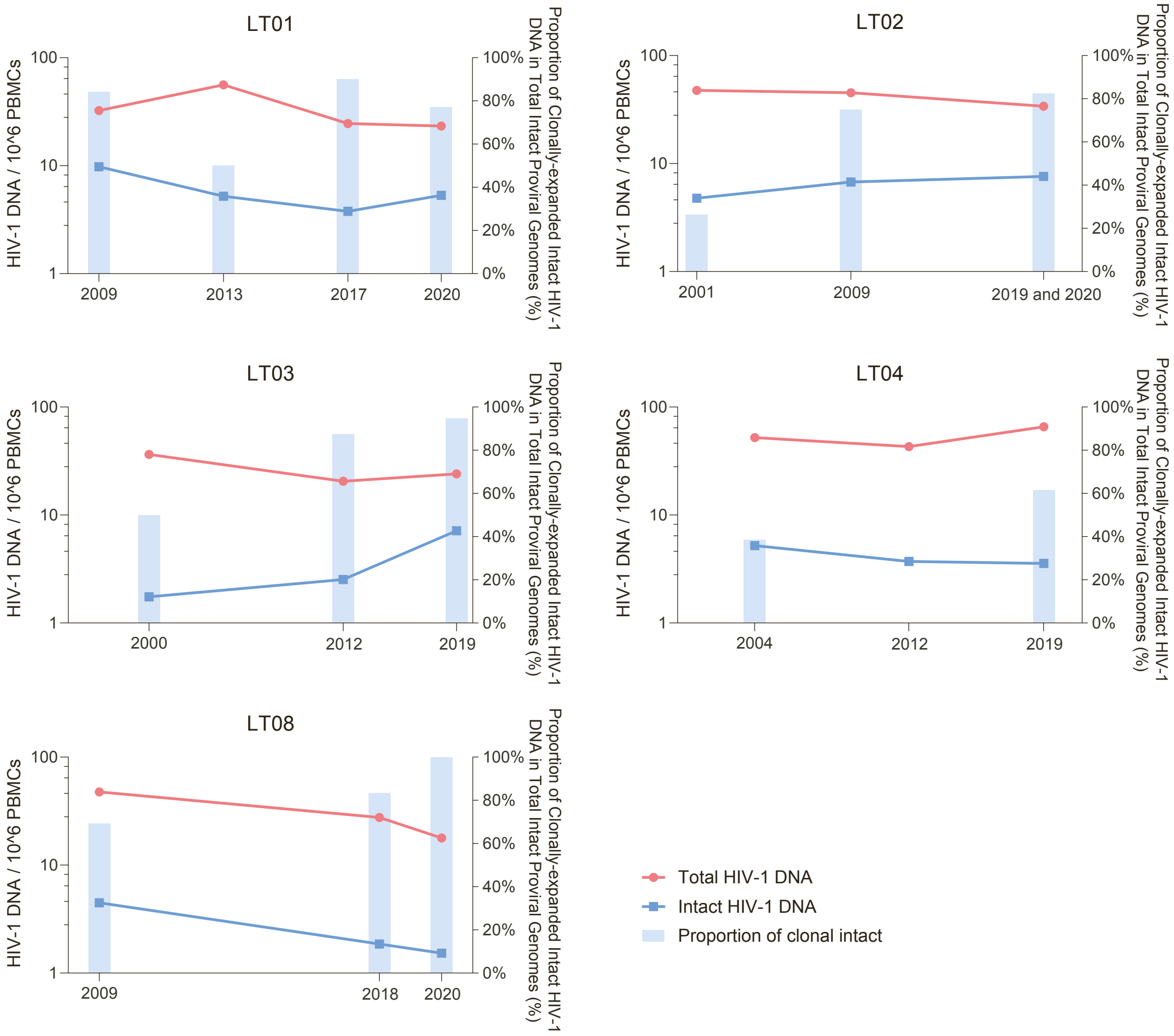

Figure S5 (Related to Figure 4): Frequencies of total and intact HIV-1 proviruses over time in the five longitudinally-followed study participants undergoing long-term ART. Proportions of clonal intact proviruses among all intact proviruses are also indicated.

Figure S6

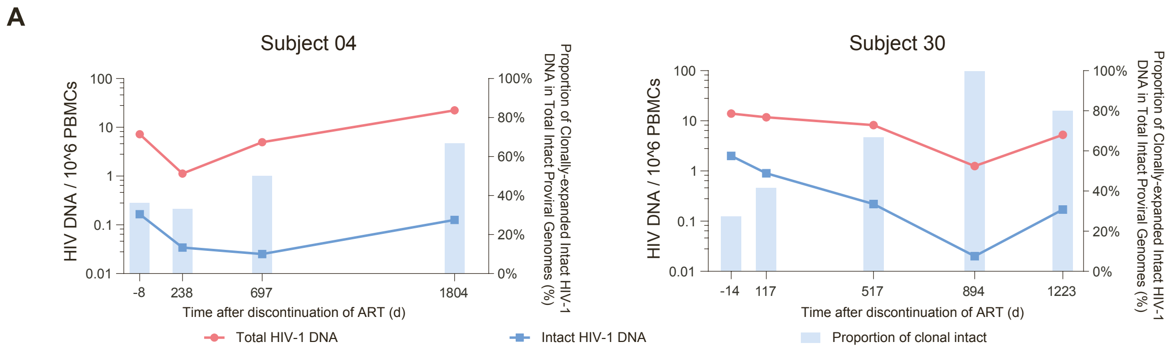

Figure S6 (Related to Figure 6): Longitudinal frequencies of HIV-1 proviruses in study persons 04 and 30. Longitudinal frequencies of total and intact HIV-1 proviruses in the 2 individuals with post-treatment control. Proportion of clonal intact proviruses among all intact proviruses is also indicated.

Table S2 (Related to Figures 2 and 4): Numbers of cells analyzed at each timepoint in indicated study participants.

Study Participant No.	Time point	Cells assayed
LT01	2009	1.94E+06
	2013	1.53E+06
	2017	2.64E+06
	2020	6.57E+06
LT02	2001	3.98E+06
	2009	1.78E+06
	2019	2.57E+06
	2020	1.15E+06
LT03	2000	2.82E+06
	2012	3.50E+06
	2019	2.66E+06
LT04	2004	1.15E+06
	2012	1.65E+06
	2019	3.64E+06
LT05	2019	8.06E+06
LT06	2021	1.27E+07
LT07	2019	8.01E+06
LT08	2009	4.85E+05
	2018	5.37E+06
	2020	2.62E+06
subject 04	on ART	1.66E+07
	d238 off ART	2.55E+07
	d697 off ART	2.43E+07
	d1804	3.61E+07
subject 30	on ART	6.60E+06
	d117 off ART	3.21E+06
	d517 off ART	5.75E+06
	d894 off ART	1.03E+07
	d1223 off ART	6.09E+06
subject 01	on ART	2.66E+07
subject 22	on ART	1.43E+06
subject 25	on ART	5.98E+06

Table S3 (Related to STAR Methods): Numbers of cells analyzed in quantitative viral outgrowth assays.

Study Participant	CD4 cells assayed	Estimated minimum number of intact proviruses based on FLIP-Seq results	Luciferase-positive wells after 21 days of culture
LT02	2.51E+06	42	1
LT03	1.93E+06	119	1
LT06	4.08E+06	20	0
LT07	1.62E+06	62	0
LT08	5.55E+06	64	0

Table S4 (Related to STAR Methods): List of Primers/Probes

Workflow	Amplicon/ Target (HXB2 Coordinates)	Round/ Orientation	Oligo Name	Oligo Sequence (5'->3')
gDNA ddPCR	LTRgag (HXB2 684-810)		LTRgag F	TCTCGACGCAGGACTCG
			LTRgag R	TACTGACGCTCTCGCACC
			LTRgag P	/56-FAM/CTCTCTCCT/ZEN/TCTAGCCTC/31ABkFQ/
	RPP30		RPP30 F	GATTTGGACCTGCGAGCG
			RPP30 R	GCGGCTGTCTCCACAAGT
			RPP30 P	/56-FAM/CTGACCTGA/ZEN/AGGCTCT/31ABkFQ/
Proviral DNA Sequencing	Near full-length (638-9632)	1F	U5-623F	AAATCTCTAGCAGTGGCGCCCGAACAG
		1R	U5-601R	TGAGGGATCTCTAGTTACCAGAGTC
		2F	U5-638F	GCGCCCGAACAGGGACYTGAAARCGAAAG
		2R	U5-547R	GCACTCAAGGCAAGCTTTATTGAGGCTTA
	Promoter (76-818)	1F	24F	CGAAGACAAGATATCCTTGATCTGTGG
		1R	962R	CTACAGCCTTCTGATGTTTCTAACAGG
		2F	76F	CTGATTAGCAGAACTACACACCAGG
		2R	818R	CCGCTTAATACTGACGCTCTCG
	Promoter (367-643)	1F	350F	GGGACTTTCCACTGGGGACTTTC
		1R	661R	GCTTTCAGGTCCCTGTTCGG
		2F	367F	ACTTTCCAGGGAGGCGTGG
		2R	Kumar R	GGGCGCCACTGCTAGAGA
	A1mod2 (638-2724)	1F	U5-623F	AAATCTCTAGCAGTGGCGCCCGAACAG
		1R	NE1	CCACTAACTTCTGTATGTCATTGACAGTCCAGCT
		2F	U5-638F	GCGCCCGAACAGGGACYTGAAARCGAAAG
		2R	ProC-	GAGTATTGTATGGATTTTCAGGCCCAAT
	Pol (2011-3798)	1F	5CP1	GAAGGGCACACAGCCAGAAATTGCAGGG
		1R	RT3.1	GCTCCTACTATGGGTTCTTTCTCTAACTGG
		2F	2.5	CCTAGGAAAAAGGGCTGTTGGAAATGTGG
		2R	RT3798R	CAAACTCCCACTCAGGAATCCA
	C (3626-5980)	1F	RT3597mixF	AAAACAGGAAARTATGCAA
		1R	SC05R	AGCTCTTCGTCGCTGTCTCCGCTT
		2F	RT3626F	TGCCCACACTAATGATGTAA
		2R	SC02R	CTTCCTGCCATAGGAGATGCCTA
	A2 (5550-7760)	1F	VP5450F	CAGGACATAACAAGGTAGGATC
		1R	CO602	GCCCATAGTGCTTCCTGCTGCTCCCAAGAACC
		2F	VP5549F	AGAGGATAGATGGAACAAGCCCCAG
		2R	V3CR	TGCTCTTTTTTCTCTCTSCACCACT
	B2 (7652-9610)	1F	GP41Fo	TTCAGACCTGGAGGAGGAGATAT
		1R	3LTRi	TCAAGGCAAGCTTTATTGAGGCTTAA
		2F	GP41Fi	GGACAATTGGAGAAGTGAATTAT
		2R	3UTRi	AGGCTTAAGCAGTGGGTTCCCTAG
Reverse Transcription	Long LTR (643)	RT	Bio Long LTR	/5BiotinTEG/AAGCAGTGGTATCAACGCAGAGTACGGGC GCCACTGCTAGAGA
	Pol (2662)	RT	Bio Pol	/5BiotinTEG/AAGCAGTGGTATCAACGCAGAGTACCAAAT TTCTACTAATGCTTTTATTTTTTC
	Nef (9040)	RT	Bio Nef	/5BiotinTEG/AAGCAGTGGTATCAACGCAGAGTACTGTAA GTCATTGGTCTTAAAGGTACCTGAGG
	PolyA (9635+25)	RT	Bio PolyA	/5BiotinTEG/AAGCAGTGGTATCAACGCAGAGTACTTTTT TTTTTTTTTTTTTTTTTTTTTTGAAG
	Tat-Rev (8459)	RT	Bio Tat-Rev	/5BiotinTEG/AAGCAGTGGTATCAACGCAGAGTACGGATC TGTCTCTGTCTCTCTCTCCACC
	Read-through (582; 9667)	RT	Bio Readth	/5BiotinTEG/AAGCAGTGGTATCAACGCAGAGTACAGAGT CACACAACAGACGG
cDNA Amplification	NA	NA	Template-switching oligo (TSO)	AAGCAGTGGTATCAACGCAGAGTACATrGrG+G-3

	NA	NA	ISPCR	AAGCAGTGGTATCAACGCAGAGT
cDNA ddPCR	Long LTR (522-643)	F	Kumar F	GCCTCAATAAAGCTTGCCTTGA
		R	Kumar R	GGGCGCCACTGCTAGAGA
		R (alternate)	625R	TTTTCCACACTGACTAAAAKGGTC
		Probe	Kumar P	/56-FAM/CCAGAGTCA/ZEN/CACAACAGACGGGCACA/3IABkFQ/
	Pol (2536-2662)	F	Pol mf299	GCACTTTAAATTTTCCCATTAGTCCTA
		R	Pol mf1	CAAATTTCTACTAATGCTTTTATTTTTTC
		Probe	Pol P	/56-FAM/AAGCCAGGA/ZEN/ATGGATGGCC/3IABkFQ/
	Nef (8883-9040)	F	F8883-03	GGTGGGAGCAGYATCTCGAGA
		R	R9040-10	TGTAAGTCATTGGTCTTAAAGGTACCTGAGG
		Probe	P8967-50	/56-FAM/CCAGGCACA/ZEN/AKCAGCATT/3IABkFQ/
	PolyA (9496-9635+25)	F	Freadth-2	GCCCTCAGATGCTRCATATAA
		R	5T25	TTTTTTTTTTTTTTTTTTTTTTTTTGAAG
		Probe	Preadth-1	/56-FAM/TGCCTGTAC/ZEN/TGGGTCTCTCTGGTTAG/3IABkFQ/
	Tat-Rev (5956-8459)	F	mf1	CTTAGGCATCTCCTATGGCAGGAA

Supplemental Reference List

[S1] Carlson, J.M., Brumme, C.J., Martin, E., Listgarten, J., Brockman, M.A., Le, A.Q., Chui, C.K., Cotton, L.A., Knapp, D.J., Riddler, S.A., et al. (2012). Correlates of protective cellular immunity revealed by analysis of population-level immune escape pathways in HIV-1. J Virol *86*, 13202-13216. 10.1128/JVI.01998-12.

[S2] Bricault, C.A., Yusim, K., Seaman, M.S., Yoon, H., Theiler, J., Giorgi, E.E., Wagh, K., Theiler, M., Hraber, P., Macke, J.P., et al. (2019). HIV-1 Neutralizing Antibody Signatures and Application to Epitope-Targeted Vaccine Design. Cell Host Microbe *26*, 296. 10.1016/j.chom.2019.07.016.

[S3] Wagner, T.A., McLaughlin, S., Garg, K., Cheung, C.Y., Larsen, B.B., Styrchak, S., Huang, H.C., Edlefsen, P.T., Mullins, J.I., and Frenkel, L.M. (2014). HIV latency. Proliferation of cells with HIV integrated into cancer genes contributes to persistent infection. Science *345*, 570-573. 10.1126/science.1256304.

[S4] Roadmap Epigenomics, C., Kundaje, A., Meuleman, W., Ernst, J., Bilenky, M., Yen, A., Heravi-Moussavi, A., Kheradpour, P., Zhang, Z., Wang, J., et al. (2015). Integrative analysis of 111 reference human epigenomes. Nature *518*, 317-330. 10.1038/nature14248.

[S5] Wagner, E.J., and Carpenter, P.B. (2012). Understanding the language of Lys36 methylation at histone H3. Nat Rev Mol Cell Biol *13*, 115-126. 10.1038/nrm3274.
